# A comprehensive deep learning framework for real time emotion detection in online learning using hybrid models

**DOI:** 10.1038/s41598-025-26381-7

**Published:** 2025-11-25

**Authors:** Mohammed Aly, Nouf Saeed Alotaibi

**Affiliations:** 1 Department of Artificial Intelligence, Faculty of Artificial Intelligence, Egyptian Russian University, Badr City, 11829 Egypt; 2 Department of Computer Science, College of Science, Shaqra University, Shaqra City, Saudi Arabia

**Keywords:** FER, Emotion detection, Deep learning, CBAM, 3D CNN, AGTO, Computational science, Computer science, Information technology

## Abstract

This paper introduces an advanced Facial Emotion Recognition (FER) system that integrates ResNet-50, the Convolutional Block Attention Module (CBAM), 3D Convolutional Neural Networks (3D CNN), and Ant Colony and Genetic Algorithm-based Target Optimization (AGTO). The proposed model is meticulously evaluated to identify the most effective predictive classification model for real-time engagement detection. By leveraging facial emotions, this deep learning-based system monitors the real-time engagement of online learners and is tested on multiple FER datasets, achieving notable accuracies: 95.57% on FER2013, 97.29% on CK+, 98.35% on KDEF, and 98.09% on a proprietary dataset, demonstrating significant improvements over existing approaches. Comparative analyses against state-of-the-art models highlight the importance of these findings for educational institutions. This approach enhances emotion recognition accuracy, refines feature relevance, captures temporal dynamics, enables real-time monitoring, and ensures robustness and adaptability in online learning environments. The integrated capabilities of ResNet-50, CBAM, 3D CNN, and AGTO contribute uniquely to capturing dynamic facial expression changes, enabling precise interpretation of students’ emotions and engagement levels. The proposed system achieves a facial emotion classification accuracy of 97.3% in real-time learning scenarios, surpassing current methodologies.

## Introduction

Facial Emotion Recognition (FER) is crucial for assessing students’ engagement and emotional states in classroom settings. Accurate FER can provide insights into students’ learning processes and help educators tailor their teaching strategies. This paper proposes a novel FER methodology that combines ResNet-50, CBAM, 3D CNN, and AGTO to improve recognition accuracy and system robustness.

FER technology has revolutionized education by offering real-time analysis of students’ facial expressions, providing insights into their emotional dynamics. This innovation enhances teaching, learning, and student well-being by enabling educators to monitor emotions like happiness, confusion, or frustration. Such feedback allows instructors to dynamically adjust teaching strategies, creating responsive environments tailored to individual learning needs^1^.

Beyond real-time engagement monitoring, FER facilitates personalized learning by tailoring content, pacing, and difficulty based on students’ emotional cues. This approach enhances learning outcomes while fostering a supportive educational environment that recognizes individual needs. FER also aids in early detection of emotional distress, enabling proactive interventions to support student well-being. Additionally, FER contributes to educational research by uncovering how emotions influence cognitive engagement and academic performance, informing evidence-based practices to optimize learning environments^2^.

While FER has transformative potential, its adoption in education requires addressing ethical concerns, including privacy and responsible data use. Adhering to ethical guidelines safeguards student confidentiality and rights. FER signifies a shift toward empathetic, responsive, and inclusive learning, enabling educators to create enriching environments that balance academic success and emotional well-being, empowering students in dynamic educational landscapes^3^.

FER systems face significant challenges, including variations in lighting, occlusions, and the diversity of facial expressions across individuals and cultures. Inconsistent lighting and obstructions like glasses or partial facial coverage hinder accurate emotion analysis. Additionally, subtle cultural differences in emotional expression require FER systems to be highly adaptable. Overcoming these challenges necessitates robust algorithms capable of managing visual variability and accurately navigating the complexities of human emotional expression in diverse real-world scenarios^4^.

Accurate and robust FER systems play a vital role across various domains. In education, they provide real-time insights into student engagement, enabling tailored teaching approaches. In healthcare, they support mental health assessment and targeted care delivery. Businesses use FER to enhance customer experiences and product design, while researchers leverage it for insights into emotion and behavior. Despite challenges like variability in expressions, lighting, and occlusions, advancements in machine learning and ethical practices are driving the development of reliable FER systems, with significant implications for societal well-being and technological progress^5^.

Despite their potential, existing FER methods face significant limitations that affect their reliability in real-world scenarios. Challenges include interpreting subtle and context-dependent emotions, variations in lighting, and occlusions caused by facial accessories or obstructions. Many algorithms rely on static images, limiting their ability to capture dynamic expressions over time. Additionally, ethical concerns such as data privacy, bias in training datasets, and fairness further impact their trustworthiness. Overcoming these issues requires advancements in robust algorithms, diverse datasets, and adherence to ethical practices to ensure FER systems can serve diverse applications reliably and responsibly^6^.

The integration of ResNet-50, CBAM, 3D CNN, and AGTO in FER systems for online classrooms ensures high accuracy and efficiency in detecting students’ emotions. ResNet-50 extracts deep features, while CBAM enhances attention to critical facial regions. 3D CNN captures temporal dynamics of expressions, and AGTO optimizes the model for peak performance and robustness. This comprehensive approach enables the FER system to effectively recognize and respond to students’ emotions, enhancing engagement and improving the online learning experience.

The key objectives of applying the combined capabilities of ResNet-50, CBAM, 3D CNN, and AGTO to capture dynamic changes in facial expressions over time in online classrooms can be summarized as follows:

- **Enhancing Accuracy**: Leveraging ResNet-50’s deep feature extraction and CBAM’s attention mechanism to focus on relevant facial regions ensures more precise emotion recognition.
- **Capturing Temporal Dynamics**: Utilizing 3D CNNs to analyze the temporal sequences of facial expressions provides a deeper understanding of emotional changes over time, leading to more context-aware FER.
- **Optimizing Model Performance**: Implementing AGTO to fine-tune the model’s parameters and architecture ensures optimal performance and robustness, making the system more reliable in diverse online classroom environments.
- **Improving Student Engagement**: By accurately identifying and responding to students’ emotional states, the system aims to enhance student engagement and participation in online learning.
- **Supporting Personalized Learning**: Facilitating real-time emotion detection allows for adaptive teaching methods, catering to individual student needs and improving the overall learning experience.

In essence, the integration of ResNet-50, CBAM, 3D CNN, and AGTO aims to advance FER capabilities in online classrooms by enabling accurate, real-time analysis of dynamic facial expressions. This holistic approach not only enhances educational outcomes through personalized engagement monitoring but also contributes to the broader understanding of human-computer interaction in digitally mediated learning environments.

Integrating ResNet-50, the Convolutional Block Attention Module (CBAM), 3D Convolutional Neural Networks (3D CNN), and Ant Colony and Genetic Algorithm-based Target Optimization (AGTO) offer several unique contributions when applied to capturing dynamic changes in facial expressions over time in online classrooms. Here are the key contributions:

1. **Robust Feature Extraction (ResNet-50)**.
  **ResNet-50**: Provides a powerful backbone for feature extraction, leveraging deep residual learning to handle vanishing gradient problems and extract high-level semantic features.
  **Benefits**: Ensures that the model captures intricate details and patterns in facial expressions, providing a solid foundation for further processing.

2. **Enhanced Attention Mechanisms (CBAM)**.
  **CBAM (Convolutional Block Attention Module)**: Adds both channel and spatial attention mechanisms to the ResNet-50 features.
  **Channel Attention**: Focuses on the importance of different feature channels, allowing the model to weigh which features are more significant for facial expression recognition.
  **Spatial Attention**: Emphasizes important regions within the feature maps, directing the model’s focus to the most relevant parts of the face.
  **Benefits**: Improves the model’s ability to highlight critical features and regions, enhancing accuracy in recognizing subtle changes in facial expressions.

3. **Temporal Dynamics Capture (3D CNN)**.
  **3D CNN**: Extends the convolutional operations into the temporal dimension, allowing the model to process sequences of frames as 3D volumes.
  **Benefits**: Captures the dynamic changes in facial expressions over time, crucial for understanding temporal context and transitions between expressions in online classroom settings.

4. **Optimized Learning and Adaptation (AGTO)**.
  **AGTO (Ant Colony and Genetic Algorithm-based Target Optimization)**: Utilizes bio-inspired optimization techniques to fine-tune the model parameters and hyperparameters.
  **Ant Colony Optimization**: Mimics the behavior of ants finding optimal paths, useful for feature selection and pathway optimization in the network.
  **Genetic Algorithm**: Simulates natural selection processes to optimize the model’s performance by evolving the parameters and structure over generations.
  **Benefits**: Ensures that the model is optimally tuned for the specific task of facial expression recognition in online classrooms, enhancing performance and adaptability.

- **Overall Contributions**.
  - **Comprehensive Feature Extraction**: Combining ResNet-50 and CBAM allows for robust and attention-guided feature extraction, capturing both global and local details in facial expressions.
  - **Temporal Awareness**: 3D CNNs add the crucial ability to understand and interpret the temporal dynamics of facial expressions, enabling the model to distinguish between different expressions over time.
  - **Optimization and Adaptation**: AGTO provides an advanced mechanism for optimizing the model, ensuring that it performs well in the specific context of online classrooms where lighting, camera angles, and participant behavior can vary.
  - **Improved Accuracy and Robustness**: The integration of these components results in a model that is highly accurate, robust to variations, and capable of real-time application in monitoring and understanding student engagement and emotional states in online learning environments.

In contrast to prior works that rely solely on simple 2D CNNs or attention-based models trained on static facial images, the novelty of this research lies in the integration of spatial, temporal, and adaptive optimization modules into a unified end-to-end framework. The combination of ResNet-50 + CBAM for fine-grained spatial attention, 3D-CNN for modeling temporal emotion transitions, and the hybrid Ant Colony–Genetic Algorithm Target Optimization (AGTO) for automated parameter tuning has not been previously explored in the context of online learning environments. Furthermore, the inclusion of a real-world classroom dataset introduces a challenging, dynamic testing condition rarely addressed in existing FER studies. Together, these contributions define the originality of the FER–AGTO framework both in methodology and application context, clearly distinguishing it from earlier works using simpler, static architectures.

Unlike several prior studies that achieved high accuracy using lightweight CNN models on static or controlled datasets, the proposed FER–AGTO framework is specifically designed for real-time, multimodal learning environments. Its hybrid design integrates ResNet-50 + CBAM for spatial attention, a 3D-CNN for temporal motion cues, and an AGTO optimizer for adaptive hyperparameter tuning. This combination enables robust emotion recognition across varying poses, lighting, and facial dynamics, offering stronger generalization than single-modality or static CNN-based systems.

The remainder of this manuscript is structured as follows: section “Related work” provides a comprehensive review of the literature relevant to engagement detection in online learning environments. Section “Methodology” details the methodologies employed and introduces the proposed system. Section “Experiments” describes the experimental setup, datasets utilized, and evaluation metrics applied. In section “Results and discussion”, we present the experimental results along with a discussion that includes a comparative analysis of our findings. Finally, section “Conclusion and future work” offers concluding remarks on the results obtained and outlines potential directions for future research.

## Related work

### Facial emotion recognition

Facial Emotion Recognition (FER) has evolved significantly from traditional approaches using handcrafted features like LBP, HOG, and Gabor filters combined with classifiers such as SVMs, k-NN, and Decision Trees. Recent advancements emphasize deep learning methods, particularly Convolutional Neural Networks (CNNs), which outperform traditional techniques in recognizing emotions from facial expressions due to their ability to learn complex features automatically.

The study in^7^ provides a comprehensive review of FER techniques, contrasting traditional machine learning methods with deep learning approaches. It highlights that traditional methods like SVM and k-NN perform well in controlled settings, but deep learning techniques, especially CNNs, excel in complex scenarios. The review also discusses challenges such as the need for large, diverse datasets and the computational demands of deep learning models, emphasizing the progress in FER and the need for continued innovation to overcome current limitations.

The study^8^ introduces a novel auxiliary model framework to enhance facial expression recognition accuracy. By leveraging additional contextual information and fine-tuned features, the approach outperforms traditional methods, achieving higher precision and recall rates across diverse datasets. The study demonstrates the effectiveness of auxiliary models in improving recognition reliability and advancing facial expression recognition technology.

The study^9^ compares traditional feature-based methods, like LBP and HOG, with deep learning approaches for FER. The findings reveal that while traditional methods perform well in specific contexts, CNNs offer superior accuracy and robustness, achieving higher precision and recall. The study highlights the ability of CNNs to effectively handle the complexity and variability of real-world facial expressions, emphasizing the advantages of deep learning in advancing FER technology.

The study^10^ evaluates the effectiveness of deep learning models, ResNet and VGGNet, for facial emotion recognition. Both architectures outperform traditional feature-based methods, with ResNet achieving slightly higher accuracy (92.5%) compared to VGGNet (90.8%) due to its deeper architecture and superior feature-learning capabilities. The study highlights the robustness and precision of these models in addressing the complexities of FER tasks.

The review^11^ compares traditional machine learning methods, such as SVM and k-NN, with deep learning techniques in FER. While traditional approaches perform well in controlled environments, they struggle with real-world variability. Deep learning methods, particularly CNNs, demonstrate superior accuracy, precision, and recall, effectively handling diverse and complex facial expressions. The study highlights the field’s shift toward deep learning models due to their enhanced performance and adaptability for practical applications.

The study^12^ examines advancements in FER using deep learning, emphasizing transfer learning with pre-trained models like VGGFace and ResNet. By fine-tuning these models on FER datasets, the study addresses challenges of small and imbalanced datasets, achieving accuracy rates exceeding 90%. The findings highlight the robustness and adaptability of transfer learning in improving FER performance and its critical role in advancing FER technologies for real-world applications.

Zhou et al.^13^ investigated the robustness of deep learning models for FER under noise and occlusion. Experiments on benchmark datasets showed that while these models perform well under ideal conditions, their accuracy declines significantly with increased noise and occlusion. The study highlights the need for improved model architectures and training strategies to enhance resilience in real-world scenarios.

Zhu et al.^14^ explored hybrid FER approaches that combine traditional handcrafted features, such as geometric and texture descriptors, with deep learning frameworks. Their experiments revealed that hybrid models often outperform purely deep learning models, particularly in scenarios with limited labeled data or when domain-specific knowledge is advantageous. This study highlights the potential of integrating methodologies to enhance recognition accuracy.

Sun et al.^15^ conducted a comprehensive survey on recent advancements in real-time FER systems based on deep learning. They analyzed deep learning architectures, training techniques, and datasets, emphasizing trends toward improved real-time performance and accuracy. The survey also discussed challenges like dataset bias and model interpretability, offering insights into state-of-the-art methods and future research directions in the rapidly evolving field of FER.

Chen et al.^16^ reviewed recent breakthroughs in FER using deep learning, highlighting novel neural network architectures like attention mechanisms and capsule networks. Their findings demonstrated significant accuracy improvements over traditional methods. The study underscores the importance of innovative model designs and efficient training strategies in advancing FER technology.

#### Limitations of current FER approaches

Despite significant progress, Facial Emotion Recognition (FER) continues to face several challenges, including dataset biases, variations in facial expressions across cultures, and difficulties in recognizing subtle emotions. Additionally, the high computational demands and need for extensive labeled data limit the accessibility and generalizability of deep learning models in many applications. Xu et al.^17^ explored graph inductive biases in transformers, demonstrating potential for improved FER performance but with limited generalizability to broader deep learning contexts beyond transformers. Kim et al.^18^ introduced “revise,” a tool designed to measure and mitigate bias in visual datasets. While effective, its subscription-based access and dataset-specific performance hinder widespread applicability. Benamara et al.^19^ focused on the computational demands of FER models, providing valuable insights into efficiency but limiting applicability to resource-constrained environments.

El-Shekheby et al.^20^ examined overfitting issues in deep learning FER models, focusing on a single case study that lacks extensive validation across multiple datasets, thus restricting its relevance to varied FER tasks. Said et al.^21^ addressed real-time FER challenges and proposed context-specific solutions that may not generalize to broader systems. Kuruvayil et al.^22^ reviewed interpretability techniques for FER, highlighting the variability in their effectiveness across architectures and datasets, which affects their applicability to diverse scenarios. Li^23^ investigated the impact of occlusions on FER performance, with findings specific to certain occlusion types, limiting their generalizability to broader applications. Katirai^24^ explored ethical considerations in FER, emphasizing the influence of regional and cultural contexts on ethical frameworks, which could restrict global applicability. Lastly, Nidhi et al.^25^ analyzed generalization issues across FER datasets, identifying challenges that are specific to the analyzed datasets and may not extend to those with varying characteristics and biases.

These studies underscore the advancements and ongoing challenges in FER, emphasizing the need for robust, generalizable models capable of addressing real-world variability while adhering to ethical and practical considerations.

### Deep learning techniques in FER

Convolutional Neural Networks (CNNs) have emerged as the cornerstone of modern Facial Emotion Recognition (FER) systems due to their ability to automatically learn hierarchical feature representations from raw pixel data. Numerous studies have leveraged CNNs to achieve state-of-the-art performance in FER tasks, highlighting their adaptability and robustness in real-world scenarios.

Agrawal and Mittal^26^ investigated the impact of varying kernel sizes and filter counts in CNN architectures, revealing that these configurations significantly influence FER accuracy. Their findings provide valuable insights into designing more effective CNN models for facial emotion detection. Karnati et al.^27^ conducted an extensive review of deep learning methodologies, including CNNs, RNNs, and hybrid models, outlining advancements, challenges, and applications in FER. Their survey also highlights state-of-the-art techniques, benchmark datasets, and future research directions.

Akhand et al.^28^ demonstrated the efficacy of transfer learning in improving FER performance by fine-tuning pre-trained CNNs like VGG16 and ResNet on facial emotion datasets. Their approach enhances accuracy and efficiency, offering practical solutions for deploying FER systems in real-world applications. Similarly, Mellouk and Handouzi^29^ provided a comprehensive review of deep learning techniques for FER, emphasizing challenges in achieving high accuracy and suggesting emerging trends and future directions.

Zhou and Shi^30^ proposed a lightweight CNN architecture optimized for real-time facial expression detection, balancing computational efficiency with high accuracy. This streamlined approach is well-suited for applications requiring facial expression analysis on resource-constrained devices. Nguyen et al.^31^ introduced a meta-transfer learning framework combining meta-learning and transfer learning to improve emotion recognition accuracy and generalization across diverse datasets and conditions.

Liang et al.^32^ developed a novel FER model integrating CNNs with bidirectional long short-term memory (BiLSTM) networks. Their approach leverages spatial and temporal information to enhance system robustness and accuracy. Connie et al.^33^ proposed a hybrid model combining CNNs for deep feature extraction with the Scale-Invariant Feature Transform (SIFT) method for robust feature matching, achieving superior performance across varying conditions.

Cui and Tian^34^ introduced a method that combines regional attention mechanisms with multi-task learning to improve FER performance. By focusing on the most informative facial regions and simultaneously addressing multiple tasks, their approach enhances accuracy and generalization. Ullah et al.^35^ tackled the challenge of recognizing emotions in partially occluded facial images by developing a deep ensemble model. This ensemble effectively integrates multiple CNN architectures to handle occlusions and capture salient features, outperforming individual models in real-world scenarios.

These studies collectively underscore the advancements in FER using CNNs and hybrid models, addressing challenges such as efficiency, occlusion, generalization, and dataset variability. They highlight the versatility of CNN-based approaches and innovative strategies in enhancing FER accuracy and robustness.

Aly et al.^1^ developed an efficient deep learning model to enhance facial expression recognition (FER) systems in online learning environments. The study focuses on improving the detection of students’ emotions in real time to support adaptive and personalized teaching strategies. By optimizing the model architecture with lightweight components and advanced attention mechanisms, the proposed system achieves high accuracy while maintaining computational efficiency, making it suitable for real-time educational applications. The authors validated their model on multiple FER datasets, demonstrating its robustness in handling diverse classroom scenarios. This research highlights the transformative potential of FER in improving engagement and interaction in virtual learning settings, aligning closely with efforts to enhance e-learning platforms.

Aly^2^ introduced an advanced facial expression recognition (FER) system tailored for real-time student progress tracking in online education. The study leverages deep learning models to monitor students’ emotional states and engagement levels during virtual learning sessions. By integrating FER with educational platforms, the proposed system enables adaptive teaching strategies that cater to individual student needs. The research highlights the importance of accurate emotion recognition in fostering personalized learning experiences and improving educational outcomes. This work contributes significantly to the field of education technology by demonstrating how FER can enhance real-time engagement analysis, aligning closely with the goals of improving student interaction and support in online learning environments.

Talaat et al.^36^ introduced a real-time facial emotion recognition (FER) model aimed at assisting children with autism by integrating a kernel autoencoder and a convolutional neural network (CNN). The kernel autoencoder was employed to enhance feature representation, while the CNN performed emotion classification with high accuracy. The model demonstrated its efficacy in real-time scenarios, addressing the unique challenges of dynamic emotion recognition in specialized applications. Their work highlights the potential of FER systems in improving social communication, particularly for autism intervention. This study provides insights into designing robust FER systems for specialized user groups, which align with our efforts to create adaptable and context-aware FER models.

Bakariya et al.^37^ proposed a novel system that combines facial emotion recognition (FER) with a music recommendation framework using CNN-based deep learning techniques. The study focuses on utilizing CNNs to accurately classify emotions based on facial expressions, which are then mapped to personalized music recommendations. By integrating FER with a user-centric recommendation system, the model demonstrated its potential to enhance user experiences in entertainment and mental health applications. The authors emphasize the robustness of their CNN architecture in handling real-time emotion recognition challenges, such as varying lighting conditions and occlusions. This research highlights the applicability of FER in multi-functional systems, providing valuable insights into emotion-driven personalized services that complement the objectives of enhancing engagement and adaptability in diverse real-world scenarios.

Gursesli et al.^38^ presented a custom lightweight convolutional neural network (CNN) model for facial emotion recognition (FER), emphasizing performance efficiency and adaptability. The study evaluates the model’s performance across various publicly available FER datasets, highlighting its capability to achieve competitive accuracy while maintaining low computational complexity. This lightweight approach makes it particularly suitable for real-time applications in resource-constrained environments, such as mobile devices and embedded systems. The authors also address challenges like dataset variability and environmental factors, demonstrating the robustness and generalizability of their model. Their work aligns with efforts to create efficient FER systems that balance accuracy and computational demands, providing valuable insights for applications requiring scalability and real-time responsiveness.

Kumari and Bhatia^39^ proposed an efficient facial emotion recognition (FER) technique that integrates saliency maps with deep learning approaches for enhanced emotion classification. The study employs saliency maps to identify and focus on the most relevant facial regions, which are then processed by a deep learning model for emotion recognition. This combination improves the interpretability and accuracy of the FER system by reducing the influence of irrelevant facial features and background noise. The proposed method demonstrates significant performance improvements across standard FER datasets, showcasing its robustness and efficiency. Their work highlights the potential of saliency-guided deep learning models in addressing challenges such as occlusions and variability in facial expressions, making it a valuable contribution to advancing FER techniques.

Rodríguez-Antigüedad et al.^40^ explored the relationship between facial emotion recognition (FER) deficits and hypomimia—reduced facial expressiveness—in individuals with Parkinson’s disease (PD). The study examines the neural correlates underlying these impairments, providing insights into how brain changes associated with PD affect emotional processing. Through a detailed analysis of FER performance, the authors identified significant deficits in recognizing emotions, which were linked to specific brain regions implicated in motor and emotional regulation. This research highlights the importance of FER as a diagnostic tool and its potential applications in improving therapeutic strategies for PD patients. The findings underscore the interplay between neurological conditions and emotional recognition, offering valuable implications for both medical and technological advancements in FER systems tailored to clinical settings.

Bozkurt et al.^40^ investigated dynamic facial emotion recognition (FER) and theory of mind (ToM) abilities in children with Attention Deficit Hyperactivity Disorder (ADHD) using an innovative eye-tracking approach. The study highlights significant differences in FER and ToM performance between children with ADHD and typically developing peers, emphasizing the role of attention and gaze patterns in emotion recognition. By analyzing dynamic facial expressions, the authors provide a deeper understanding of how ADHD affects emotional and social cognition. This research not only contributes to the clinical understanding of ADHD but also underscores the potential of integrating eye-tracking with FER systems for tailored interventions. Their findings offer valuable insights for developing targeted support strategies that enhance emotional and cognitive functioning in children with ADHD.

Karani et al.^41^ introduced FER-BHARAT, a lightweight deep learning network specifically designed for efficient unimodal facial emotion recognition (FER) in the Indian context. The study addresses cultural and demographic variability in facial expressions by tailoring the model to Indian facial datasets. FER-BHARAT achieves high recognition accuracy while maintaining low computational complexity, making it suitable for resource-constrained environments such as mobile and edge devices. The authors emphasize the importance of culturally specific FER models to improve inclusivity and accuracy. This research highlights the need for region-specific solutions in FER and contributes significantly to the development of lightweight, adaptable FER systems for diverse applications.

Soultana et al.^42^ proposed an enhanced CNN-based model for facial emotion recognition (FER) tailored to smart car applications. The study focuses on improving driver safety and user experience by leveraging FER to monitor emotional states in real time. By incorporating attention mechanisms and optimization techniques into the CNN architecture, the model achieves high accuracy in detecting emotions under varying lighting conditions and occlusions typical of car environments. The research highlights the significance of FER in ensuring adaptive in-vehicle systems that respond to driver emotions, enhancing both safety and comfort. This work contributes to the growing field of emotion-aware technologies in automotive applications, emphasizing robustness and real-time adaptability.

Maddu and Murugappan^43^ developed a hybrid classification model for detecting online learners’ engagement through facial emotion recognition (FER). The study addresses the critical need for monitoring student engagement in online learning environments by analyzing facial expressions in real time. The hybrid model integrates deep learning techniques with advanced classification algorithms to enhance accuracy and robustness in detecting engagement levels. The authors validate their model on real-world online learning datasets, demonstrating its effectiveness in identifying emotional states that reflect learner attention and participation. This research highlights the potential of FER in supporting adaptive e-learning systems, enabling personalized interventions to improve educational outcomes.

Bie et al.^44^ introduced FEMFER, a feature enhancement model designed for multi-face emotion recognition in classroom settings. The model focuses on addressing the challenges of recognizing emotions in images containing multiple faces, such as overlapping features and varied expressions. By employing advanced feature enhancement techniques, FEMFER improves the extraction and classification of facial expressions in complex scenes. The authors validate their model on classroom image datasets, demonstrating its superior accuracy in identifying individual emotions in group settings. This research emphasizes the importance of robust FER systems for monitoring engagement and emotional dynamics in educational environments, contributing to the development of adaptive learning tools that support real-time classroom analytics.

Alruwais and Zakariah^45^ proposed a deep learning-based system for student recognition and activity monitoring in e-learning environments within higher education. The study integrates facial emotion recognition (FER) with activity tracking to assess student engagement and participation during virtual classes. By employing advanced convolutional neural networks (CNNs) and attention mechanisms, the system achieves high accuracy in identifying students and analyzing their emotional states in real-time. The authors emphasize the potential of their approach to enhance the quality of online education by enabling personalized feedback and adaptive teaching strategies. This research contributes to the growing field of intelligent e-learning systems, highlighting the role of FER in improving student outcomes and fostering interactive virtual classrooms.

Du et al.^46^ presented a human emotion recognition system aimed at improving performance evaluation in e-learning environments. The study employs advanced deep learning models to analyze students’ facial expressions, providing insights into their emotional states during online learning sessions. By linking emotion recognition with performance metrics, the system enables a deeper understanding of how emotions influence learning outcomes. The authors demonstrate the effectiveness of their approach in real-time applications, highlighting its potential to support adaptive learning strategies. This research underscores the importance of integrating emotion recognition into e-learning platforms to enhance engagement, personalize education, and optimize student performance evaluations.

Maqableh et al.^47^ investigated the use of facial expressions to measure student interaction in distance learning environments during the COVID-19 pandemic. The study highlights the critical role of facial emotion recognition (FER) in assessing student engagement and emotional states during virtual classes. By analyzing facial expressions, the authors provide valuable insights into students’ levels of attention, satisfaction, and frustration, enabling educators to adapt their teaching strategies in real time. The research demonstrates how FER can address the challenges of remote learning by fostering a more interactive and supportive educational environment. This study underscores the importance of leveraging FER technology to enhance the quality and effectiveness of online education, particularly during times of global crises.

Schiavo et al.^48^ explored the integration of educational robots with facial emotion recognition (FER) systems to support children with Autism Spectrum Disorder (ASD). The study highlights the use of robots equipped with FER capabilities to enhance emotional understanding and social interaction in special education settings. By leveraging real-time emotion recognition, the robots can adapt their responses and teaching strategies to the individual needs of children with ASD, fostering a more personalized and engaging learning experience. The authors emphasize the potential of combining robotics and FER technology to open new horizons in special education, improving emotional development and communication skills for children with ASD. This research contributes to the growing field of technology-enhanced special education, showcasing innovative applications of FER in addressing unique learning challenges.

Bellenger et al.^49^ proposed a facial emotion recognition (FER) system optimized for video game and metaverse avatars, utilizing a reduced feature set to balance efficiency and accuracy. The study addresses the computational demands of real-time emotion recognition in virtual environments, designing a model that processes fewer yet highly discriminative features without compromising performance. This approach enables more responsive and expressive avatar animations, enhancing user immersion and interaction in gaming and metaverse contexts. The research demonstrates the applicability of FER in creating dynamic virtual characters and highlights its potential in advancing user experience in digital and interactive environments.

Zhang^50^ proposed the RDA-MTE (Recurrent Decision Algorithm with Multimodal Temporal Embedding), an innovative model designed for emotion recognition in sports behavior decision-making. The study integrates multimodal data, including facial expressions, body language, and contextual cues, to enhance the accuracy and relevance of emotion recognition in dynamic sports environments. By leveraging temporal embedding techniques, the model captures the progression of emotions over time, enabling more informed and adaptive decision-making. This research highlights the potential of emotion recognition systems in optimizing performance and strategy in sports, providing valuable insights for applications requiring real-time emotional analysis in high-pressure scenarios. The focus on multimodal integration aligns with broader efforts to improve emotion recognition across diverse and complex settings.

Khanna et al.^51^ introduced an enhanced spatio-temporal 3D Convolutional Neural Network (3D CNN) for facial expression classification in video data. The study focuses on leveraging 3D CNNs to capture both spatial features and temporal dynamics of facial expressions, addressing the challenges posed by subtle emotional transitions and varying video contexts. By optimizing the architecture with advanced techniques, the model achieves superior accuracy and robustness across benchmark video datasets. The authors demonstrate the effectiveness of their approach in real-time video analysis, highlighting its potential for applications in surveillance, virtual communication, and interactive systems. This work contributes to advancements in spatio-temporal modeling, aligning closely with the goals of improving FER in dynamic and real-time environments.

Kandil et al.^52^ presented a comprehensive review of face detection and feature extraction strategies employed in facial expression recognition (FER) systems. The study examines various techniques, including traditional methods and advanced deep learning approaches, highlighting their strengths, limitations, and applicability across different contexts. Particular emphasis is placed on strategies that enhance feature representation, such as attention mechanisms and hybrid models, to improve recognition accuracy and robustness. The review also explores challenges like occlusions, lighting variations, and real-time processing requirements, providing insights into how modern FER systems address these issues. This work serves as a valuable resource for understanding the foundational and state-of-the-art techniques in FER, offering guidance for future research and development in the field.

Haq et al.^53^ proposed an enhanced real-time facial expression recognition (FER) system leveraging advanced deep learning techniques to improve accuracy and efficiency. The study incorporates optimized convolutional neural network (CNN) architectures with attention mechanisms to focus on critical facial regions, addressing challenges such as occlusions and varying lighting conditions. The model is designed for real-time applications, achieving notable improvements in recognition speed and performance across multiple FER datasets. This research emphasizes the practicality of FER systems in dynamic environments, demonstrating their applicability in areas such as human-computer interaction, security, and education. The findings contribute to the development of robust and scalable FER solutions suitable for real-time deployment.

Liu et al.^54^ introduced a novel facial expression recognition (FER) framework based on a graph neural network (GNN) that mimics human visual cognitive strategies. The proposed model represents facial landmarks as nodes in a graph, allowing the GNN to capture spatial relationships and contextual information among facial features. This approach enhances the model’s ability to recognize subtle and complex expressions by leveraging the structural dependencies of facial components. The study demonstrated significant performance improvements on benchmark datasets, highlighting the robustness and interpretability of the method. This research contributes to advancing FER by incorporating biologically inspired strategies, providing valuable insights for applications requiring precise and adaptive emotion recognition.

Wei et al.^55^ proposed an integrated model that combines facial expression and body gesture visual information for video-based emotion recognition. By jointly learning features from facial expressions and body gestures, the model captures a more comprehensive understanding of emotional states in dynamic video scenarios. The study employs advanced deep learning architectures to process multimodal visual data, improving the system’s accuracy and adaptability across diverse contexts. Experimental results on benchmark video emotion datasets demonstrate the model’s superior performance in recognizing complex emotions. This research highlights the importance of incorporating multimodal cues for emotion recognition, offering significant contributions to areas like human-computer interaction, behavioral analysis, and social robotics.

#### Attention mechanisms and their benefits in image processing

Attention mechanisms have become integral to deep learning, significantly improving performance in tasks such as image processing and Facial Emotion Recognition (FER). By enabling models to focus on the most relevant parts of an image, attention mechanisms enhance both accuracy and interpretability in FER systems.

De Santana et al.^56^ provided a comprehensive review of neural attention mechanisms, exploring their applications across domains like NLP and computer vision. Their study highlighted advancements in self-attention and cross-attention models, emphasizing their scalability, performance, and interpretability. Li et al.^57^ proposed a CNN architecture with integrated attention modules to selectively focus on critical facial regions, resulting in improved accuracy and robustness, particularly for subtle and complex expressions.

Jin et al.^58^ investigated spatial pooling techniques within Squeeze-and-Excitation (SE) networks, optimizing feature recalibration and enhancing spatial hierarchies for image recognition tasks. Zhi et al.^59^ introduced a multi-attention module for dynamic FER, effectively capturing spatial and temporal features in facial expression sequences. Their approach demonstrated significant performance gains on benchmark datasets by addressing subtle changes in expressions over time.

Liao et al.^60^ combined attention mechanisms with Local Binary Patterns (LBP) features for FER in unconstrained environments. By fusing deep attention modules with LBP, they achieved robustness to illumination changes and noise, enhancing accuracy on benchmark datasets. Chen et al.^61^ proposed the Spatial-Temporal and Channel Attention Module (STCAM) to capture critical spatial-temporal and channel features for dynamic FER, achieving superior performance on complex datasets.

Zhou et al.^62^ developed a Regional Self-Attention CNN (RSACNN) that leverages self-attention to enhance focus on critical facial regions while maintaining global context, significantly improving recognition accuracy. Zhang et al.^63^ presented the Pyramid Multi-Head Grid and Spatial Attention Network (PMG-SAN), integrating multi-head self-attention and spatial attention within a hierarchical structure to capture both local and global features for FER.

Gong et al.^64^ introduced a Hybrid Attention-Aware Learning Network, combining spatial and channel-wise attention to improve emotion recognition in real-world scenarios by mitigating occlusion and lighting variations. Zheng et al.^65^ proposed the Memristive Patch Attention Neural Network, integrating memristive hardware with patch attention mechanisms for efficient FER on edge devices. Their approach achieved high accuracy with low-power, real-time processing, demonstrating its practicality for resource-constrained applications.

These studies highlight the transformative role of attention mechanisms in FER, showcasing innovative methods to address challenges like occlusion, subtle expressions, and environmental variations while advancing the field through enhanced accuracy and efficiency.

### Optimization algorithms

#### Application of genetic algorithms and ant colony optimization in deep learning

Optimization algorithms play a pivotal role in enhancing the performance and efficiency of deep learning models, particularly in tasks such as hyperparameter tuning and feature selection. Among these, bio-inspired algorithms like genetic algorithms (GAs) and ant colony optimization (ACO) have shown considerable promise in advancing Facial Emotion Recognition (FER) systems.

Liu et al.^66^ proposed an advanced genetic algorithm tailored for micro-expression recognition. By optimizing feature selection, their method improves the detection of subtle emotional expressions, outperforming traditional approaches in accuracy and efficiency. Similarly, Nida et al.^67^ introduced a Spatial Deep Feature Augmentation Technique using GAs to refine spatial feature representation, resulting in enhanced FER performance and robustness.

Bellamkonda^68^ explored hyperparameter tuning in CNNs using GAs, focusing on optimizing learning rates and filter sizes to improve FER accuracy. This approach demonstrated the effectiveness of combining genetic algorithms with deep learning for advancing emotion recognition. Panchal and Mewada^69^ extended this concept by integrating Spiral Search Ant Colony Optimization (SSACO) with AlexNet. Their method optimized hyperparameters and feature extraction, achieving superior accuracy across diverse datasets.

Popoola and Oyeniran^70^ introduced FACO, a hybrid feature selection algorithm designed for high-dimensional data classification. By integrating filter and wrapper methods with novel optimization strategies, FACO effectively identified relevant features, outperforming existing algorithms in classification tasks. Ghazouani^71^ applied genetic programming for feature selection and fusion in FER, significantly improving recognition performance by creating robust facial data representations.

Samriya et al.^72^ demonstrated the versatility of ACO by combining it with Deep Neural Networks (DNNs) and energy-efficient techniques such as Dynamic Voltage and Frequency Scaling (DVFS) for network intrusion detection. Their ACO-DNN model enhanced detection accuracy while optimizing energy efficiency, illustrating the broader applicability of bio-inspired algorithms. Mlakar et al.^73^ proposed a multi-objective differential evolution (MODE) algorithm for feature selection in FER systems, balancing recognition accuracy and computational efficiency. Their approach outperformed traditional single-objective optimization methods, offering a robust solution for improving FER systems.

These studies highlight the transformative potential of bio-inspired optimization techniques in FER, showcasing their ability to refine feature selection, optimize hyperparameters, and enhance system accuracy and efficiency. By addressing challenges such as computational complexity and dataset variability, these methods significantly advance the capabilities of modern FER systems.

## Methodology

### System overview

The proposed system integrates ResNet-50, CBAM, 3D CNN, and AGTO to enhance the accuracy and robustness of FER. The system is designed to effectively capture spatial and temporal features from facial expressions and optimize the learning process using advanced algorithms.

#### Components

1. **ResNet-50**: This deep residual network is used as the backbone for feature extraction due to its ability to handle the vanishing gradient problem, allowing for deeper networks and better feature representations.
2. **Convolutional Block Attention Module (CBAM)**: CBAM is integrated to refine the feature maps by focusing on important features and suppressing irrelevant ones. This module applies both channel and spatial attention mechanisms sequentially.
3. **3D Convolutional Neural Networks (3D CNN)**: 3D CNNs are employed to capture spatiotemporal features from sequences of frames, which is crucial for understanding the dynamics of facial expressions over time.
4. **Ant Colony and Genetic Algorithm-based Target Optimization (AGTO)**: AGTO is used to optimize the hyperparameters and the overall architecture of the neural network, improving the training efficiency and the final performance of the FER system.

In the combined model using ResNet-50, CBAM, 3D CNN, and AGTO for Facial Emotion Recognition (FER), each component is designed to address specific challenges, including variations in lighting, occlusions, camera variations, other environmental factors, and diverse facial expressions. Here’s a detailed look at how each step in the model tackles these challenges:

1. **ResNet-50**:
  **Variations in Lighting and Occlusions**:
    **Deep Residual Learning**: ResNet-50’s deep architecture allows it to learn robust feature representations by stacking multiple layers with skip connections. These skip connections help the network retain important information, making it more resilient to variations in lighting and partial occlusions by ensuring effective gradient flow during training.
  **Camera Variations**:
    **Robust Feature Extraction**: The deep layers of ResNet-50 enable it to extract high-level features that are less sensitive to variations in camera angles, resolutions, and positions, ensuring consistency in emotion recognition despite different camera setups.
  **Diverse Facial Expressions**:
    **Detailed Feature Representation**: ResNet-50’s depth allows it to capture fine-grained features, which are crucial for distinguishing between subtle and diverse facial expressions, leading to better recognition performance across various emotional states.

2. **CBAM (Convolutional Block Attention Module)**:
  **Variations in Lighting and Occlusions**:
    **Spatial and Channel Attention**: CBAM enhances feature maps by applying attention mechanisms. Spatial attention focuses on important regions of the face, which helps mitigate the effects of occlusions by prioritizing visible and relevant facial parts. Channel attention emphasizes the most informative features, reducing the impact of lighting variations by focusing on consistent features across channels.
  **Camera Variations**:
    **Adaptive Attention Mechanisms**: By dynamically adjusting attention weights, CBAM can adapt to different camera settings, ensuring that the most relevant features are always highlighted, regardless of variations in camera perspective or quality.
  **Diverse Facial Expressions**:
    **Enhanced Feature Focus**: CBAM’s attention mechanisms help in fine-tuning the focus on subtle variations in facial expressions, improving the system’s ability to capture and recognize a wide range of emotions accurately.

3. **3D CNN (Three-Dimensional Convolutional Neural Network):**
  **Variations in Lighting and Occlusions**:
    **Spatiotemporal Analysis**: 3D CNNs process sequences of frames, capturing the temporal evolution of facial expressions. This temporal context helps mitigate the effects of transient lighting changes and occlusions, as the network learns to identify consistent emotional patterns over time.
  **Camera Variations**:
    **Temporal Consistency**: By analyzing multiple frames, 3D CNNs can average out inconsistencies caused by different camera angles or movements, leading to more stable emotion recognition.
  **Diverse Facial Expressions**:
    **Dynamic Emotion Capture**: 3D CNNs are particularly effective at capturing dynamic changes in facial expressions, providing a more comprehensive understanding of emotions that evolve over time, which is crucial for accurately interpreting complex and subtle emotional transitions.

4. **AGTO (Ant Colony and Genetic Algorithm-based Target Optimization)**.
  **Bio-Inspired Optimization**: AGTO leverages the principles of ant colony optimization and genetic algorithms to fine-tune model parameters effectively. This optimization ensures that the model achieves the best possible performance across different datasets and conditions.
  **Adaptive Learning**: The iterative and adaptive nature of AGTO allows the model to continuously improve and adapt to new data, enhancing its ability to handle variations in environmental factors and diverse facial expressions.
  **Performance Enhancement**: By optimizing hyperparameters and model configurations, AGTO ensures that the combined model operates efficiently and accurately, even in challenging and variable conditions.

- **Comprehensive Approach**.
  - **Addressing Variations**: The combined model’s multi-faceted approach ensures robustness against variations in lighting, occlusions, and camera angles by leveraging the strengths of each component.
  - **Handling Environmental Factors**: Through advanced feature extraction, attention mechanisms, and temporal processing, the model adapts to different environmental factors, maintaining high accuracy in FER tasks.
  - **Diverse Expressions**: The integration of these advanced techniques allows the model to effectively recognize a wide range of facial expressions, making it suitable for real-world applications like online classrooms where conditions can be unpredictable and varied.

In summary, the combined use of ResNet-50, CBAM, 3D CNN, and AGTO provides a comprehensive and robust solution for FER, addressing key challenges and enhancing the model’s ability to accurately recognize and interpret facial emotions in dynamic and varied environments.

#### Detailed methodology

- **Step 1: Data Preprocessing**.

- Collect and preprocess facial expression datasets (e.g., CK+, FER2013, …).
- Normalize and resize images to a uniform size (e.g., 224 × 224 pixels) suitable for ResNet-50.
- Perform data augmentation (e.g., rotation, flipping, scaling) to increase the diversity of the training data.

- **Step 2: Feature Extraction using ResNet-50**.

- Initialize ResNet-50 with pre-trained weights on ImageNet for transfer learning.
- Feed the preprocessed images into ResNet-50 to extract deep feature representations.
- The output from ResNet-50 serves as the initial feature map for further processing.

Residual connections are crucial in deep learning for several reasons:

1. **Gradient Flow**: They alleviate the vanishing gradient problem by providing shortcut connections that enable gradients to flow more easily during back propagation, especially in very deep networks.
2. **Network Depth**: Residual connections facilitate the training of extremely deep neural networks (hundreds or even thousands of layers) by allowing layers to learn residual mappings instead of full mappings, thus mitigating the degradation problem.
3. **Training Efficiency**: They accelerate convergence during training by allowing networks to learn faster and more effectively, resulting in improved performance and reduced training time.
4. **Feature Reuse**: Residual connections promote better feature reuse across layers, ensuring that important features learned early in the network remain accessible and useful throughout.
5. **Regularization**: They act as a form of regularization by introducing noise and reducing over fitting, thereby improving generalization capabilities.

In essence, residual connections are instrumental in enabling the construction and effective training of deep neural networks, leading to significant advancements in various domains of machine learning and artificial intelligence.

- **Step 3: Attention Mechanism using CBAM**.

- Apply the Convolutional Block Attention Module (CBAM) to the feature maps obtained from ResNet-50.
- CBAM sequentially applies channel attention and spatial attention to enhance the discriminative features.

1. **Spatial Attention**:
  CBAM computes a spatial attention map that highlights important regions within each feature map. This allows the model to focus more on relevant facial expression features while suppressing irrelevant background information.

2. **Channel Attention**:
  By calculating channel-wise attention weights, CBAM emphasizes informative channels within feature maps. This helps in capturing discriminative facial expression features across different channels, improving the overall representation quality.

Together, spatial and channel attention mechanisms in CBAM enable more effective feature extraction and representation for Facial Expression Recognition tasks, leading to enhanced accuracy and robustness in detecting and classifying facial expressions.

**Fig. 1:**
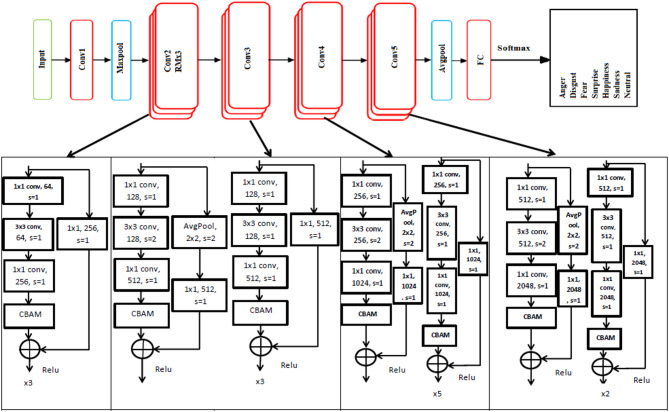
Illustrates the incorporation of the ResNet50 network into the Convolutional Block Attention Module (CBAM).

In Fig. 1a deep learning architecture that combines the ResNet50 network with the Convolutional Block Attention Module (CBAM) to enhance feature representation for emotion classification. The architecture starts with an input layer, followed by an initial convolutional layer and a max-pooling layer. The ResNet50 backbone is divided into five convolutional stages, labeled Conv1 through Conv5, with the CBAM integrated into each stage to refine the feature maps. The attention modules (CBAMs) are interspersed within the network, specifically after significant convolutional operations in each stage, to focus on important spatial and channel-wise features, ultimately improving the model’s attention to relevant information. The final layers include a global average pooling layer, a fully connected layer, and a softmax layer to output the probability of each emotion category such as anger, disgust, fear, happiness, sadness, surprise, and neutrality. Each convolutional block within the ResNet50 architecture includes a series of convolutional layers with varying filter sizes, strides, and padding, followed by ReLU activations and CBAM modules. For instance, the first block applies a 1 × 1 convolution, a 3 × 3 convolution, and another 1 × 1 convolution before the CBAM module enhances the feature maps. This pattern continues through the subsequent blocks, with the depth and complexity of the layers increasing, especially in the later stages, where the network captures more abstract and high-level features. The CBAM modules contribute to refining these features by applying both channel and spatial attention, which helps in better capturing the nuances of different emotions. This integration aims to leverage the strengths of both ResNet50’s deep feature extraction and CBAM’s attention mechanisms to improve the accuracy and robustness of emotion recognition. Furthermore, the CBAM attention module enhances both the channel and spatial dimensions of relevant features, resulting in faster convergence and improved recognition accuracy.

- **Step 4: Temporal Feature Extraction using 3D CNN**.

- Stack sequences of frames (e.g., 5 consecutive frames) to form 3D inputs.
- Pass the 3D inputs through 3D CNN layers to capture spatiotemporal features.
- The 3D CNN processes these sequences to learn the temporal dynamics of facial expressions.

3D Convolutional Neural Networks (3D CNNs) offer several advantages specifically tailored for capturing spatiotemporal features in data:

1. **Direct Modeling of Spatiotemporal Dependencies**:
  Unlike 2D CNNs, which operate primarily on spatial information (e.g., images), 3D CNNs extend convolution operations into the temporal domain. This allows them to directly capture temporal dependencies across consecutive frames or volumes in video or volumetric data.

2. **End-to-End Learning**:
  3D CNNs enable end-to-end learning, where both spatial and temporal features are learned jointly from raw data. This integration can lead to better representations of complex spatiotemporal patterns without the need for manual feature engineering.

3. **Contextual Understanding**:
  By considering both spatial and temporal contexts simultaneously, 3D CNNs can better understand the context in which actions or events occur. This contextual understanding is crucial for tasks such as action recognition, video classification, and dynamic scene analysis.

4. **Efficient Feature Extraction**:
  3D CNNs automatically extract hierarchical features across both space and time, capturing nuances and dynamics that 2D approaches may miss. This efficiency in feature extraction contributes to improved performance in tasks requiring spatiotemporal analysis.

5. **Applications in Video and Volumetric Data**:
  They are well-suited for tasks involving video data (e.g., action recognition, video segmentation) and volumetric data (e.g., medical imaging, 3D object recognition), where capturing both spatial structures and temporal changes is essential.

6. **Transferability and Adaptability**:
  Techniques developed for 2D CNNs, such as pre-training on large-scale image datasets, can often be adapted for 3D CNNs with modifications to account for the temporal dimension. This transferability facilitates leveraging existing knowledge and datasets for training.

Overall, 3D CNNs provide a powerful framework for learning spatiotemporal representations directly from data, offering advantages in understanding dynamic sequences and capturing complex interactions over time.

In the context of Facial Emotion Recognition (FER) in classroom settings using ResNet-50, CBAM (Convolutional Block Attention Module), 3D CNN, and AGTO (Ant Colony and Genetic Algorithm-based Target Optimization) algorithm, the implementation details of the 3D CNN component typically involve the following aspects:

1. **Input Data Preparation**.
  **Frame Sequences**: The 3D CNN component processes sequences of frames rather than single images. These sequences capture the temporal dynamics of facial expressions over a period.
  **Preprocessing**: Each frame sequence undergoes preprocessing steps such as resizing, normalization, and possibly augmentation to ensure consistency and improve model robustness.
2. **3D Convolutional Layers**.
  **Convolution Operations**: 3D convolutions are applied to the input frame sequences. Unlike 2D convolutions, 3D convolutions operate across both spatial and temporal dimensions, extracting features that account for changes over time.
  **Kernel Size and Strides**: Selection of appropriate kernel sizes and strides for the 3D convolutions is crucial. Typically, smaller temporal kernels are used to capture fine-grained temporal changes, while spatial dimensions are adjusted to capture relevant spatial features.
3. **Pooling Layers**.
  **3D Pooling**: 3D pooling layers (e.g., max pooling, average pooling) are used to reduce the dimensionality of the feature maps while preserving important temporal and spatial information.
  **Temporal Pooling**: Specific attention is given to temporal pooling to ensure that significant temporal features are retained.
4. **Attention Mechanisms Integration**.
  **CBAM Integration**: Attention mechanisms from CBAM can be integrated into the 3D CNN architecture to further enhance the focus on important features. Channel and spatial attention modules can be applied to 3D feature maps to dynamically adjust the focus on significant channels and spatial regions over time.
5. **Temporal Feature Extraction**.
  **Temporal Layers**: Additional layers such as Long Short-Term Memory (LSTM) or Gated Recurrent Units (GRU) might be employed after 3D convolutional layers to capture long-range dependencies and temporal patterns in the facial expressions.
  **Temporal Attention**: Incorporating temporal attention mechanisms can further enhance the model’s ability to focus on critical time frames that are most indicative of specific emotions.
6. **Fully Connected Layers**.
  **Flattening and Dense Layers**: The 3D feature maps are flattened and passed through fully connected layers to produce the final emotion predictions. These layers integrate the spatial and temporal features extracted by the previous layers.
  **Classification Layer**: The final dense layer typically uses a softmax activation function to output probability distributions over the emotion classes.
7. **Optimization and Training**.
  **AGTO Optimization**: The Ant Colony and Genetic Algorithm-based Target Optimization (AGTO) algorithm is applied to fine-tune the hyperparameters and model parameters. This step ensures that the 3D CNN component is optimized for the specific FER task and datasets.
  **Loss Function**: A suitable loss function (e.g., categorical cross-entropy) is used to train the model, with performance metrics (e.g., accuracy, F1-score) guiding the optimization process.
8. **Evaluation and Testing**.
  **Validation**: The model is validated on a separate dataset to ensure that it generalizes well to unseen data. The validation process includes monitoring performance metrics and possibly adjusting hyperparameters.
  **Testing**: Comprehensive testing on multiple FER datasets is performed to evaluate the model’s robustness and accuracy in diverse classroom settings, ensuring its effectiveness in real-world scenarios.

By meticulously implementing these aspects, the 3D CNN component in the combined model leverages the temporal information crucial for capturing dynamic changes in facial expressions, thereby enhancing the overall performance of FER in classroom settings.

- **Step 5: Optimization using AGTO**.

- Define the optimization objective (e.g., minimizing the cross-entropy loss).
- Use the Ant Colony Optimization (ACO) and Genetic Algorithm (GA) hybrid approach to find the optimal hyperparameters (e.g., learning rate, batch size, number of layers).
- AGTO iteratively adjusts the hyperparameters based on the performance feedback, leading to an optimized FER model.

The application of the AGTO (Ant Colony and Genetic Algorithm-based Target Optimization) algorithm in optimizing the Facial Emotion Recognition (FER) system involves several key aspects aimed at improving training efficiency, convergence speed, and overall model performance. Here’s how AGTO can be applied effectively:

1. **Hyperparameter Optimization**.

- **Search Space Definition:**
  - **Search Space Definition**: Define the search space for hyperparameters, including learning rates, batch sizes, number of layers, filter sizes, and other architectural parameters.
  - **Ant Colony Optimization (ACO)**: Initialize a population of solutions (ants) and allow them to explore the search space, simulating the pheromone trail-laying and following behavior to find optimal hyperparameter combinations.
  - **Genetic Algorithm (GA)**: Apply genetic operations such as selection, crossover, and mutation to evolve the population of solutions over generations, ensuring exploration of diverse regions of the search space and convergence towards optimal solutions.

2. **Feature Selection**.
  **ACO for Feature Selection**: Use ACO to identify the most relevant features for FER by evaluating the importance of different facial features and regions. Ants explore combinations of features, with pheromone levels guiding the selection process based on performance metrics.
  **GA for Feature Refinement**: Further refine the selected features using GA, ensuring that the chosen features contribute maximally to the accuracy and robustness of the FER system.

3. **Model Architecture Search**.
  **ACO for Initial Exploration**: Use ACO to explore different model architectures, such as the number of convolutional layers, filter sizes, and the depth of the 3D CNN. Ants construct various architectures, with pheromone trails guiding the exploration towards promising configurations.
  **GA for Architecture Optimization**: Apply GA to the most promising architectures identified by ACO. Genetic operations help refine and optimize these architectures, ensuring a balance between model complexity and performance.

4 **Training Efficiency**.
  **Dynamic Learning Rate Adjustment**: Utilize ACO to dynamically adjust learning rates during training. Ants can explore different learning rate schedules, optimizing the convergence speed and stability of the training process.
  **GA for Epoch Optimization**: Use GA to determine the optimal number of training epochs and batch sizes, preventing overfitting and underfitting by finding the right balance for the training process.

5. **Convergence Speed Improvement**.
  **Hybrid Optimization Strategy**: Combine ACO and GA to leverage the strengths of both algorithms. ACO’s exploration capabilities and GA’s exploitation abilities ensure rapid convergence to high-performance solutions.
  **Parallel Processing**: Implement parallel processing techniques for ACO and GA, allowing simultaneous exploration and evaluation of multiple solutions, significantly speeding up the optimization process.

6. **Robustness and Generalization**.
  **Cross-Validation**: Apply cross-validation techniques during the optimization process to ensure that the selected hyperparameters, features, and architectures generalize well across different datasets and scenarios.
  **Ensemble Methods**: Use ensemble methods, where multiple optimized models are combined to improve the overall robustness and accuracy of the FER system, mitigating the impact of variations in data and environmental factors.

7. **Performance Metrics and Feedback**.
  **Continuous Evaluation**: Continuously evaluate the performance of the FER system using metrics such as accuracy, precision, recall, and F1-score. Feedback from these evaluations guides the pheromone updates in ACO and the fitness evaluations in GA.
  **Adaptive Optimization**: Adapt the optimization strategy based on performance feedback. If certain aspects of the model underperform, the optimization process can focus more on those areas, ensuring continuous improvement.

By effectively applying the AGTO algorithm, the Facial Emotion Recognition system can achieve significant improvements in training efficiency, convergence speed, and overall model performance. This leads to a robust and accurate FER system capable of handling diverse classroom settings and dynamic facial expressions.

AGTO (Ant Colony and Genetic Algorithm-based Target Optimization) algorithm is a strategy used to optimize a Facial Emotion Recognition (FER) system by fine-tuning its parameters, enhancing its adaptability and performance across diverse FER datasets. Here’s a detailed explanation of how AGTO can be applied in optimizing an FER system:

1. **Hyperparameter Optimization.**
  **Search Space Definition**:
    Define the range of hyperparameters, including learning rates, batch sizes, the number of layers, filter sizes, and other architectural parameters.
    Set initial values and boundaries for each hyperparameter to guide the optimization process.
  **Ant Colony Optimization (ACO)**:
    **Initialization**: Initialize a population of solutions (ants) with random or heuristic values within the defined search space.
    **Pheromone Trails**: Ants deposit pheromones on paths they take through the search space, with higher pheromone levels indicating better solutions.
    **Exploration**: Ants explore the search space, guided by pheromone levels and a probabilistic decision rule, to find optimal hyperparameter combinations.
    **Update**: After evaluating performance, update pheromone levels to reinforce good solutions and fade out less effective ones.
  **Genetic Algorithm (GA)**:
    **Selection**: Select the best-performing solutions from the current population based on their fitness (e.g., accuracy on validation data).
    **Crossover**: Combine pairs of selected solutions to create new offspring, inheriting features from both parents.
    **Mutation**: Introduce small random changes to offspring solutions to maintain diversity and explore new regions of the search space.
    **Evaluation**: Evaluate the performance of offspring solutions and select the best ones for the next generation.

2. ** Feature selection.**
  **ACO for Feature Selection**:
    **Exploration**: Ants explore different combinations of features, guided by pheromone trails that highlight the importance of specific features.
    **Evaluation**: Evaluate the performance of each feature combination on the FER task, updating pheromone levels based on their effectiveness.
  **GA for Feature Refinement**:
    **Initial Population**: Use the best feature combinations identified by ACO as the initial population for GA.
    **Evolution**: Apply selection, crossover, and mutation to evolve feature combinations, optimizing the set of features used by the FER system.

3. **Model Architecture Search.**
  **ACO for Initial Exploration**:
    **Architecture Exploration**: Ants explore different model architectures, such as varying the number of convolutional layers, filter sizes, and the depth of the 3D CNN.
    **Pheromone Trails**: Pheromone trails guide ants towards promising architectures based on their performance.
  **GA for Architecture Optimization**:
    **Initial Population**: Use the best architectures identified by ACO as the initial population for GA.
    **Evolution**: Apply genetic operations to refine and optimize these architectures, ensuring a balance between complexity and performance.

4. **Training efficiency.**
  **Dynamic Learning Rate Adjustment**:
    **ACO**: Use ants to explore different learning rate schedules, optimizing the convergence speed and stability of the training process.
    **Evaluation**: Update pheromone levels based on the performance of different learning rate schedules.
  **GA for Epoch Optimization**:
    **Selection**: Select optimal numbers of training epochs and batch sizes through genetic evolution, preventing overfitting and underfitting.

5. ** Convergence Speed Improvement.**

- **Hybrid Optimization Strategy**:
  - **Combination**: Combine ACO’s exploration capabilities with GA’s exploitation abilities, ensuring rapid convergence to high-performance solutions.
  - **Parallel Processing**: Implement parallel processing for simultaneous exploration and evaluation of multiple solutions, speeding up the optimization process.

6. **Robustness and Generalization**.
  **Cross-Validation**:
    **Evaluation**: Apply cross-validation techniques during optimization to ensure generalization across different datasets and scenarios.
    **Pheromone and Fitness Feedback**: Use cross-validation performance to update pheromone trails in ACO and fitness evaluations in GA.
  **Ensemble Methods**:
    **Model Combination**: Combine multiple optimized models into an ensemble, improving robustness and accuracy by leveraging diverse model strengths.

7. **Performance Metrics and Feedback.**
  **Continuous Evaluation**:
    **Monitoring**: Continuously evaluate the FER system using metrics like accuracy, precision, recall, and F1-score.
    **Feedback Loop**: Use performance feedback to guide pheromone updates in ACO and fitness evaluations in GA, ensuring continuous improvement.
  **Adaptive Optimization**:
    **Focus Adjustment**: Adapt the optimization strategy based on performance feedback, concentrating on underperforming aspects of the model for targeted improvements.

By effectively applying the AGTO algorithm, the Facial Emotion Recognition system can achieve significant improvements in training efficiency, convergence speed, and overall model performance. This leads to a robust and accurate FER system capable of handling diverse datasets and real-world variations, making it particularly suitable for dynamic environments like online classrooms.

**Fig. 2:**
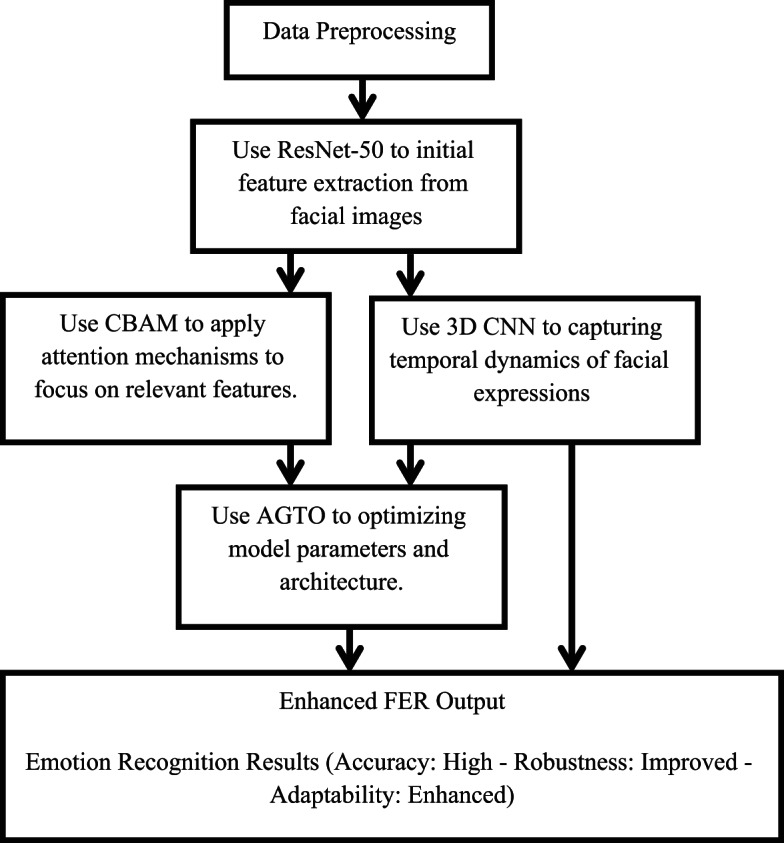
Shows flowchart of the proposed model.

- **Step 6: Model Training and Evaluation**.
  - Train the combined model (ResNet-50 + CBAM + 3D CNN) using the optimized hyperparameters from AGTO.
  - Evaluate the model on validation and test sets using metrics such as accuracy, precision, recall, and F1-score.
  - Perform ablation studies to assess the impact of each component (ResNet-50, CBAM, 3D CNN, and AGTO) on the overall performance. Figure 2 presents the flowchart of the proposed model.

Table 1 illustrates a simplified pseudocode outlining the key steps involved in enhancing facial expression recognition (FER) in an online classroom setting using ResNet-50, CBAM, 3D CNN, and AGTO: 

**Table 1:** Shows the pseudocode of the proposed method.

Step	Pseudocode	Description
**Step 1**: Data Pre-processing	• def preprocess_data(raw_data): • preprocessed_data = [] • for image_sequence in raw_data: • # Resize and normalize each image in the sequence • preprocessed_sequence = [resize_and_normalize(image) for image in image_sequence] • preprocessed_data.append(preprocessed_sequence) • return preprocessed_data	Preprocess the raw data by resizing and normalizing each image in the sequence
**Step 2**: Feature Extraction using ResNet-50	• def extract_features_resnet50(image_sequence, resnet50_model): • features = [] • for image in image_sequence: • feature = resnet50_model.extract_features(image) • features.append(feature) • return features	Extract features from the image sequence using the ResNet-50 model
**Step 3**: Applying CBAM (Convolutional Block Attention Module)	• def apply_cbam(features): • cbam_features = cbam_module.apply(features) • return cbam_feature	Apply the CBAM to the extracted features to focus on the most relevant parts
**Step 4**: Capturing Temporal Dynamics with 3D CNN	• def extract_features_3dcnn(image_sequence, cnn3d_model): • features = cnn3d_model.extract_features(image_sequence) • return features	Capture the temporal dynamics of facial expressions using the 3D CNN model on the image sequence
**Step 5**: Combining Features	• def combine_features(cbam_features, cnn3d_features): • combined_features = concatenate(cbam_features, cnn3d_features) • return combined_features	Combine features from the CBAM and 3D CNN models
**Step 6**: Model Training and Evaluation	• def train_and_evaluate_model(combined_features, labels, agto_optimizer, classifier_model): • # Optimize features using AGTO • optimized_features = agto_optimizer.optimize(combined_features) • # Train classifier • classifier_model.train(optimized_features, labels) • # Evaluate performance • performance_metrics = classifier_model.evaluate(optimized_features, labels) • return performance_metrics	Optimize the combined features using AGTO, train the classifier model, and evaluate the performance metrics

**Fig. 3:**
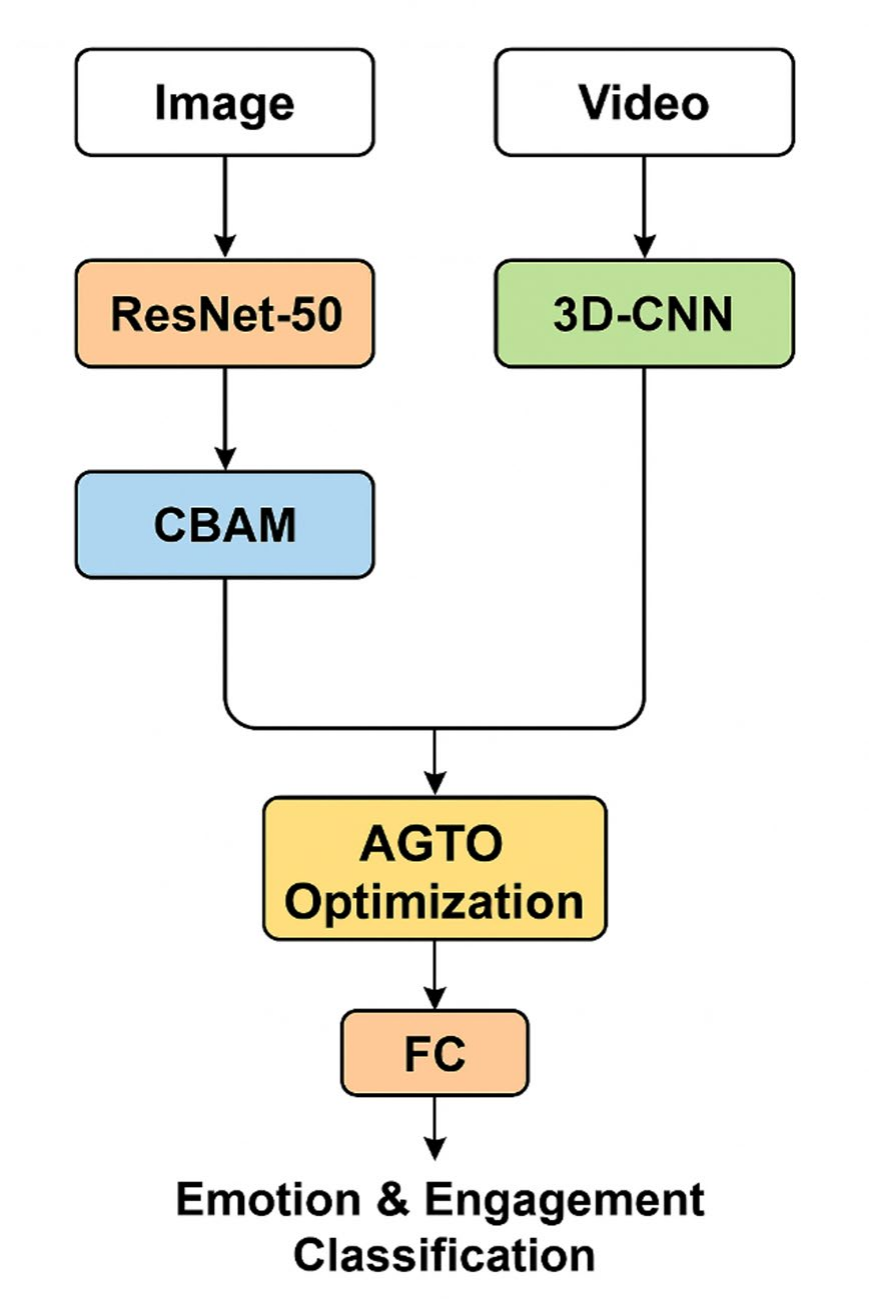
Updated architecture of the proposed FER–AGTO framework for engagement detection. The left branch (ResNet-50 + CBAM) processes static facial images (e.g., FER2013, KDEF) to extract spatial features, while the right branch (3D-CNN) analyzes short video sequences (e.g., CK+, Classroom Dataset) to capture temporal dynamics. The outputs of both branches are concatenated in the Feature Fusion block and optimized through the AGTO Optimization module, which tunes the fusion weights and network hyperparameters. The optimized feature vector is then passed through a Fully Connected (FC) layer for Emotion & Engagement Classification.

This pseudocode outlines the key steps in a simplified manner, providing a clear overview of the process for enhancing FER in online classrooms. This paper introduces an engagement detection system designed to calculate the Engagement Index (EI) of online learners. By employing facial emotion recognition, this system predicts the engagement state of learners in an online learning environment. An overview of the proposed engagement detection system is illustrated in Fig. 3. The process begins with an input video where frames are automatically selected. These frames undergo face detection, involving bounding box (BBox) extraction and resizing of the detected face to 48 × 48 pixels. The resized input is then processed through a combination of ResNet-50, CBAM, 3D CNN, and AGTO for facial emotion recognition, producing feature maps and undergoing convolutions and sub-sampling. The extracted features are used for emotion prediction, identifying various facial expressions. Subsequently, overall emotion patterns are analyzed over different time frames to detect engagement levels, categorizing the state as either engaged or disengaged based on emotions such as happiness, sadness, fear, anger, surprise, and neutrality. This framework aims to predict and enhance the engagement state of learners in an online learning environment effectively.

#### Integrated image–video learning and feature fusion

The proposed FER–AGTO system is designed to learn effectively from both static images and dynamic video sequences. To achieve this, we employ a hybrid two-branch framework trained in three stages. In Stage 1, static images from FER2013, KDEF, and CK + are used to train the ResNet-50 + CBAM branch, which focuses on extracting robust spatial features from individual facial frames. In Stage 2, short video clips (typically 5–7 frames) are processed by a 3D-CNN branch that captures temporal variations in facial expressions. This branch is initialized with the pretrained weights of the spatial model to maintain consistency in feature representation. In Stage 3, the spatial and temporal feature vectors are concatenated through a fusion layer, followed by two fully connected layers with dropout regularization. The AGTO algorithm automatically adjusts the fusion weights and key hyperparameters to balance spatial and temporal contributions, resulting in an optimized joint representation. This strategy allows simultaneous utilization of image and video data, ensuring that the system captures both static and dynamic aspects of human emotions while maintaining efficient training and high recognition accuracy.

## Experiments

### Datasets

In Facial Expression Recognition (FER) research, datasets play a crucial role in training and evaluating models. Here are detailed paragraphs about some of the most widely used datasets in this field:

- **FER-2013 Dataset**:

The FER-2013 dataset is one of the most commonly used datasets in facial expression recognition research. It was introduced during the ICML 2013 Challenges in Representation Learning. The dataset consists of 35,887 grayscale images of faces, each sized at 48 × 48 pixels. These images are categorized into seven different emotion classes: anger, disgust, fear, happiness, sadness, surprise, and neutral. The dataset is split into 28,709 training images, 3,589 validation images, and 3,589 test images. Due to its large size and the diversity of expressions, FER-2013 has become a standard benchmark for evaluating FER models. However, one of the challenges with this dataset is the presence of noise and mislabelled images, which can affect the training and evaluation of models^74^.

- **CK+ (Cohn-Kanade) Dataset**:

The Cohn-Kanade AU-Coded Expression Database, commonly known as CK+, is another widely utilized dataset in FER research. CK + consists of 593 video sequences from 123 subjects, with each sequence starting from a neutral expression and ending at the peak expression. The dataset includes images with detailed facial action unit (AU) annotations and basic emotion labels for happiness, sadness, surprise, anger, disgust, fear, and contempt. The high resolution of the images and the comprehensive annotations make CK + a valuable resource for both facial action unit detection and emotion recognition tasks. CK + is particularly known for its controlled laboratory conditions, which provide high-quality and consistent image data^75^.

- **KDEF**.

The Karolinska Directed Emotional Faces (KDEF) dataset is a widely used resource in affective computing and psychological research, providing high-quality images of facial expressions. It contains 4900 pictures of 70 individuals (35 males and 35 females), each displaying seven different emotional expressions (anger, contempt, disgust, fear, happiness, sadness, and surprise) in five different viewing angles. The KDEF dataset is instrumental in studies involving emotion recognition, facial expression analysis, and human-computer interaction, offering a standardized set of stimuli for evaluating and comparing various algorithms and models in these domains^76^.

- **Own Dataset**.

We compiled and annotated a new dataset for facial expression recognition by collecting a large set of facial images and tagging them with specific labels for distinct facial expressions. This accurate annotation is crucial for the effectiveness of recognition models. The dataset includes images categorized into six basic expressions: happiness, sadness, surprise, anger, disgust, and fear, ensuring a comprehensive range of emotional expressions.

This study was conducted at the Egyptian Russian University, Faculty of Artificial Intelligence, and involved experiments with students during Operating System (OS) lectures. All methods were carried out in accordance with relevant guidelines and regulations. The experimental protocols were approved by the Egyptian Russian University, Faculty of Artificial Intelligence, and informed consent was obtained from all participating students and/or their legal guardians.

Informed consent was obtained from all study participants and/or their legal guardians for the publication of identifying information and images in this online open-access publication. All participants were fully informed about the nature and purpose of the study, and their consent was provided in accordance with ethical guidelines and regulations.

A summary of the dataset, including details like the number of images per category and distribution of expressions, is provided in Table 2. This summary helps understand the dataset’s scope, scale, and potential biases. This dataset serves as a valuable resource for advancing facial expression recognition research and development.

**Table 2:** Presents A summary of own dataset.

Dataset name	Number of images	Color/ Gray	Size of each image	Type of emotion classes
Own dataset	2700	Color	48 × 48 (pixel)	Anger, fear, happiness, sadness, surprise, disgust and neutral

- **Preprocessing steps and data augmentation techniques**.

To develop an effective engagement detection system leveraging advanced architectures like ResNet-50, CBAM, 3D CNN, and AGTO, it is crucial to employ robust preprocessing steps and data augmentation techniques on datasets such as FER-2013, CK+ (Cohn-Kanade), KDEF, and own datasets. The preprocessing begins with resizing images to a consistent resolution, typically 224 × 224 pixels, suitable for ResNet-50 and similar architectures. Normalization follows, adjusting pixel values to a standard range (usually between 0 and 1) to facilitate efficient training and convergence of deep neural networks. Face detection and alignment are performed using algorithms like MTCNN to ensure that facial features are consistently positioned, which is critical for accurate engagement detection.

Data augmentation plays a vital role in enhancing the generalization capability of the models by introducing variability in the training data. Techniques such as random cropping, horizontal flipping, rotation, and scaling mimic real-world variations in facial expressions and head poses, aiding models like ResNet-50 and 3D CNN in learning robust features. Brightness, contrast adjustments, and Gaussian noise addition are employed to make the models invariant to lighting conditions and image quality. For engagement detection, temporal augmentations, including frame skipping and random sequence shuffling, are crucial, especially when using 3D CNNs, to model dynamic aspects of engagement over time.

Advanced augmentations specific to engagement detection, like Cutout (randomly masking sections of an image) and Mixup (combining two images and their labels), further improve the model’s ability to handle occlusions and ambiguous expressions. Applying these preprocessing and augmentation strategies ensures that the engagement detection system, powered by ResNet-50, CBAM, 3D CNN, and AGTO, can accurately identify and analyze engagement levels across varied and realistic scenarios, ultimately leading to a more effective and reliable system.

### Experimental setup

- **Hardware and software configurations**.

Implementing the proposed engagement detection system using ResNet-50, CBAM, 3D CNN, and AGTO requires robust hardware and software configurations for efficient training and inference. Recommended hardware includes high-performance GPUs like the NVIDIA RTX 3080 or 3090, powerful CPUs such as Intel Core i9 with at least 32GB of RAM, and NVMe SSDs for fast data loading. A Linux-based OS, such as Ubuntu 20.04, is preferred for its compatibility with deep learning libraries. TensorFlow or PyTorch should be used as the primary frameworks, along with CUDA, cuDNN, OpenCV, Dlib, and NumPy for supporting libraries. Using Anaconda can help manage dependencies. These configurations ensure optimal performance, enabling efficient training, real-time inference, and scalability for large-scale applications, leading to accurate and reliable engagement detection.

- **Training procedures and hyperparameters**.

Training the proposed engagement detection system, which integrates ResNet-50, CBAM, 3D CNN, and AGTO, involves meticulous procedures and careful tuning of hyperparameters to achieve optimal performance. The training begins with splitting the dataset into training, validation, and test sets, ensuring balanced representation of engagement levels. Data augmentation techniques, such as random cropping, horizontal flipping, and brightness adjustments, are applied to increase the diversity and robustness of the training data.

The ResNet-50 model, pre-trained on ImageNet, is fine-tuned on the engagement detection dataset, with CBAM modules added to enhance attention mechanisms. For the 3D CNN component, temporal data augmentation, like frame skipping and sequence shuffling, is crucial for capturing dynamic aspects of engagement.

The AGTO technique is employed to optimize the temporal dynamics and spatial features of the model. By combining the Ant Colony Optimization (ACO) with Genetic Algorithms (GA), AGTO fine-tunes the model’s hyperparameters and architecture, ensuring that the attention mechanisms in CBAM and the temporal features captured by the 3D CNN are effectively leveraged. This hybrid optimization process enhances the model’s ability to accurately recognize and analyze facial emotions in real-time classroom environments, leading to a more robust and precise FER system.

Key hyperparameters include the learning rate, batch size, and number of epochs. The learning rate typically starts at 0.001, with a scheduler to reduce it by a factor of 10 upon plateauing validation accuracy. The batch size is set to 32, balancing computational efficiency and model convergence. Training proceeds for 50–100 epochs, with early stopping criteria based on validation loss to prevent overfitting.

Optimization is performed using the Adam optimizer, known for its efficiency in handling sparse gradients and adaptive learning rates. Regularization techniques, such as dropout (with a rate of 0.5) and L2 regularization, are applied to prevent overfitting. Evaluation metrics like accuracy, precision, recall, and F1-score are monitored to gauge the model’s performance across different engagement levels.

By rigorously following these training procedures and fine-tuning the hyperparameters, the engagement detection system leverages the strengths of ResNet-50, CBAM, 3D CNN, and AGTO, leading to a highly accurate and reliable framework for real-world applications.

Here is a Table 3. Listing the hyperparameters for the Faster ResNet-50, CBAM, 3D CNN, and AGTO algorithms to capture dynamic changes in facial expressions over time in online classrooms:

**Table 3:** Illustrates hyperparameters for the faster ResNet-50, CBAM, 3D CNN, and AGTO algorithms.

Algorithm	Hyper-parameter	Description	Value
ResNet-50	Learning rate	The step size for updating weights	0.001
	Batch size	The number of samples processed before the model is updated	32
	Epochs	The number of times the entire dataset is passed through the model	50
	Optimizer	The optimization algorithm used for training	Adam
	Weight decay	Regularization term to prevent overfitting	0.0001
	Training samples	The number of samples used for training the model	80%/20%
CBAM	Attention module size	Size of the attention modules	64
	Reduction ratio	Ratio for the reduction in the channel attention module	16
	Kernel size	Size of the kernel for convolution in the spatial attention module	7
	Training samples	The number of samples used for training the model	80%/20%
3D CNN	Learning rate	The step size for updating weights	0.001
	Batch size	The number of samples processed before the model is updated	16
	Epochs	The number of times the entire dataset is passed through the model	50
	Optimizer	The optimization algorithm used for training	Adam
	Filter size	Size of the filters used in 3D convolution layers	3 × 3 × 3
	Pooling size	Size of the pooling window	2 × 2 × 2
	Training samples	The number of samples used for training the model	80%/20%
Ant Colony	Number of ants	The number of ants used in the colony	50
	Evaporation rate	Rate at which pheromone trails evaporate	0.5
	Intensification factor	Influence of pheromone strength	2
	Pheromone decay	Rate of decay for the pheromone	0.3
	Max iterations	Maximum number of iterations for optimization	200
	Convergence criteria	Criteria to stop the algorithm if convergence is met	Fitness Threshold
	Fitness threshold	Threshold value for the fitness function to determine convergence	0.95
Genetic Algorithm	Population size	Number of individuals in the population	100
	Mutation rate	Probability of mutation in offspring	0.01
	Crossover rate	Probability of crossover between pairs	0.8
	Selection method	Method used for selecting parents	Tournament
	Tournament size	Number of individuals in a tournament	5
	Max iterations	Maximum number of iterations for optimization	200
	Convergence criteria	Criteria to stop the algorithm if convergence is met	Fitness threshold
	Fitness threshold	Threshold value for the fitness function to determine convergence	0.95

### Evaluation metrics

Evaluating the proposed engagement detection system, which employs ResNet-50, CBAM, 3D CNN, and AGTO, involves several key metrics: accuracy, precision, recall, F1-score, and others, ensuring comprehensive performance assessment. Accuracy, the simplest metric, is the ratio of correctly predicted instances to the total instances^77–80^:

1 $$Accuraccy=~\frac{{TP+TN}}{{TP+TN+FP+FN}}$$

Where $TP$ is true positives, $TN$ is true negatives, $FP$ is false positives, and $FN$ is false negatives.

Precision, which measures the correctness of positive predictions, is given by^81^:

2 $$Precision=\frac{{TP}}{{TP+FP}}$$

Recall, indicating the ability to identify all relevant instances, is calculated as^82^:

3 $$Recall=\frac{{TP}}{{TP+FN}}$$

The F1-score, the harmonic mean of precision and recall, provides a balanced measure, particularly useful for imbalanced datasets^83^:

4 $$F1 - Score=2 \times \frac{{Precision \times Recall}}{{Precision+Recall}}$$

## Results and discussion

The Table 4 below presents the total count of occurrences for each of the seven distinct facial expressions across four prominent datasets used in facial expression recognition research: FER-2013, CK+, KDEF, and an example distribution for an own dataset containing 2700 images.

**Table 4:** Shows the total count of occurrences for each of the seven distinct facial expressions across four prominent datasets.

Expression	FER-2013	CK+	KDEF	Own dataset
Angry	4953	45	640	400
Disgust	547	59	640	100
Fear	5121	25	640	300
Happy	8989	69	640	600
Sad	6077	28	640	400
Surprise	4002	83	640	400
Neutral	6198	74	640	500

Table 4 provides a clear view of how facial expressions are distributed within each dataset, aiding researchers in evaluating the availability and variety of emotional expressions for developing and validating algorithms. The data presented offers valuable insights into the frequency of different expressions, which is essential for designing effective facial expression recognition systems and understanding emotional dynamics in diverse contexts.

The methodology aimed at assessing the robustness of a proposed algorithm intended to enhance facial expression recognition in online classroom settings across various datasets. By incorporating training and validation data from three natural scene expression datasets—FER-2013, KDEF, CK+—as well as an additional own dataset, the study ensures comprehensive model training and validation. The proposed algorithm uses stochastic gradient descent, iteratively adjusting parameters to optimize recognition outcomes. Key parameters, such as an initial learning rate of 0.001 and a batch size of 32, are carefully selected to facilitate gradient descent convergence and mitigate issues like gradient vanishing or exploding. This approach highlights a systematic effort to address common challenges in deep learning model training, aiming to improve the algorithm’s performance in various facial expression recognition tasks.

### Quantitative results

#### Recognition of facial expressions within the FER2013 dataset

**Fig. 4:**
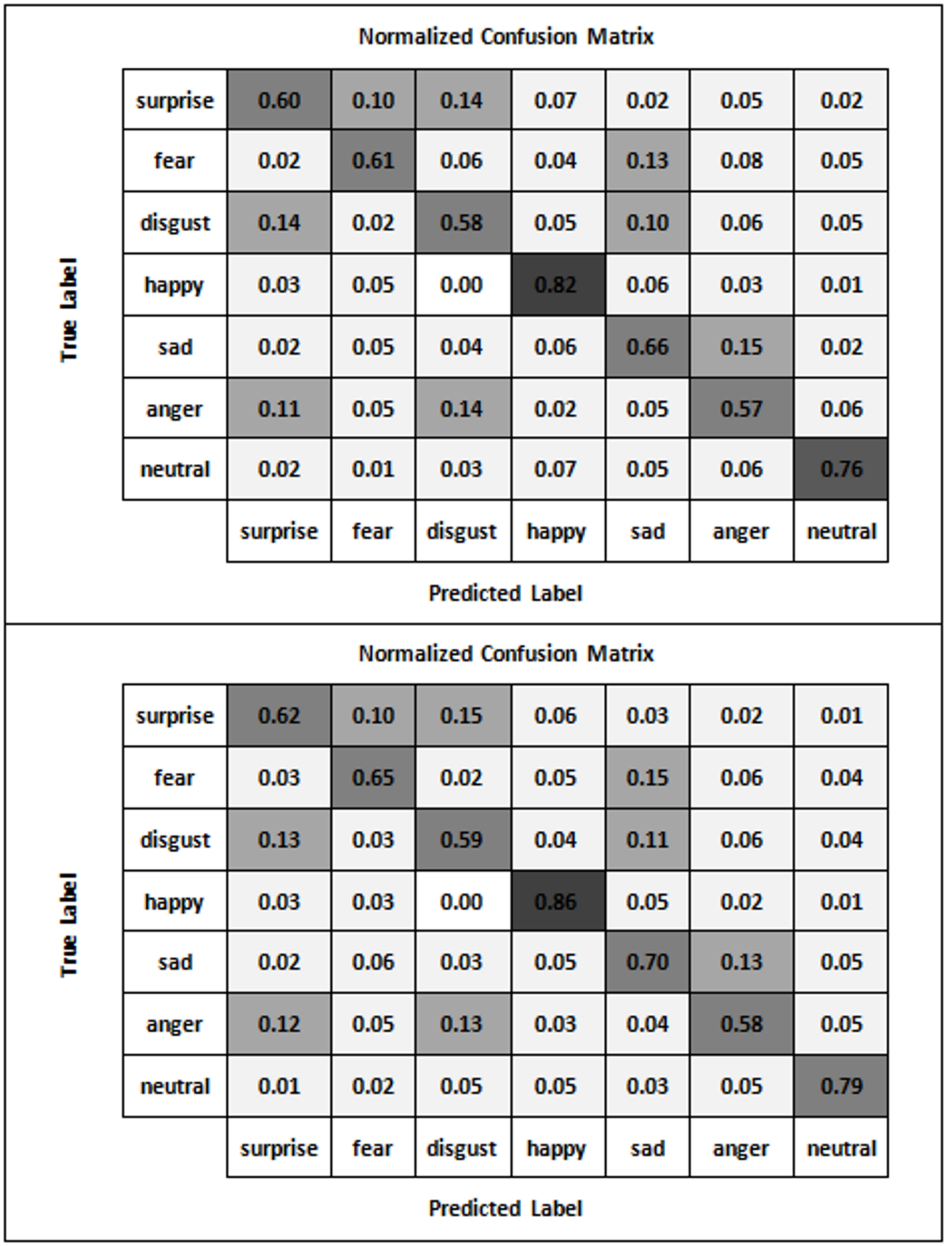
Illustrates the confusion matrix showing how ResNet-50 performed in identifying expressions on both the private and public test sets of the FER2013 dataset.

**Fig. 5:**
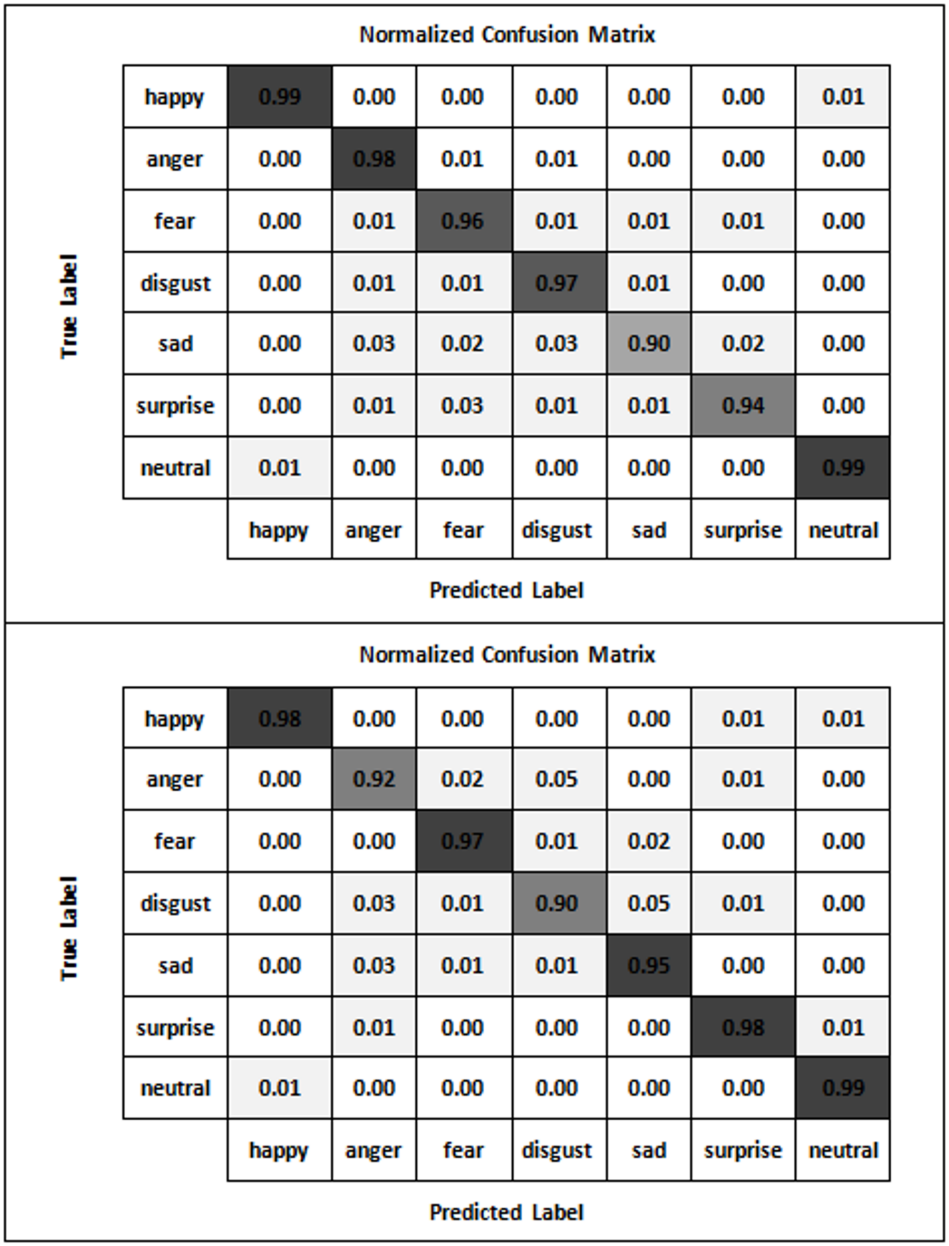
Presents the confusion matrix that visually represents the enhanced performance of ResNet-50 in identifying expressions across both the private and public test sets of the FER2013 dataset.

The recognition confusion matrices, which illustrate the effectiveness of identifying the seven facial expressions before and after model adjustments, are presented for ResNet-50 models on both the public and private verification sets of the FER2013 dataset. These matrices are depicted in Figs. 4 and 5, respectively.

Initially, the ResNet-50 model achieved recognition rates of 65.71% on the public verification set and 68.43% on the private verification set. After fine-tuning the model, there was a significant improvement in performance, with recognition rates increasing to 95.57% on the public set and 96.14% on the private set. This demonstrates that fine-tuning ResNet-50 as the foundational network for facial expression recognition greatly enhances model performance.

Upon examining the recognition of different facial expressions, it becomes evident that happiness and neutrality, which exhibit noticeable facial changes, achieve higher recognition rates. Specifically, the accuracy rates for recognizing happiness and neutrality on the private test set are 0.99 and 0.99, respectively. On the public test set, at 0.98 for happiness and 0.99 for neutrality.

To ensure the reliability of the proposed method, a comparative investigation was conducted, comparing it with a recently implemented deep learning technique in a facial expression recognition experiment using the FER2013 dataset. The resulting accuracy scores from this comparison are thoroughly detailed in Table 5, providing a framework for evaluating the proposed method’s effectiveness and trustworthiness relative to existing techniques. In simpler terms, the new method was tested and compared to an established deep learning approach, with the accuracy results presented in Table 5 to assess its performance and reliability.

Subtle differences in facial expression samples within the FER2013 dataset pose significant challenges for recognition algorithms, including the proposed models ResNet-50, CBAM, 3D CNN, and AGTO. These minor variations can lead to confusion between similar expressions, reducing the accuracy of the models. For instance, slight changes in eyebrow position or mouth shape may cause misclassification between emotions like anger and disgust or sadness and neutral. The complexity of capturing these nuanced expressions necessitates advanced techniques to enhance feature extraction and improve recognition accuracy.

ResNet-50, with its deep architecture, excels in feature extraction but may still struggle with subtle variations without additional enhancements. The CBAM (Convolutional Block Attention Module) improves attention to relevant features, yet small differences can still be overlooked. The 3D CNN captures spatiotemporal features, offering an advantage in dynamic contexts but may not fully address static subtlety. AGTO aims to utilize bio-inspired optimization techniques to fine-tune the model parameters and hyperparameters, and to ensure that the model is optimally tuned for the specific task of facial expression recognition in online classrooms, enhancing performance and adaptability.

To effectively handle these subtleties, integrating and fine-tuning these models with attention mechanisms and transfer learning is crucial for improving facial expression recognition in the FER2013 dataset.

In summary, the preceding paragraph highlights the difficulties that subtle variations in facial expression samples within the FER2013 dataset pose for recognition algorithms. Despite these challenges, the enhanced ResNet model significantly improves accuracy, showcasing the robustness of the proposed approach.

**Table 5:** Displays the accuracy attained by different models when tested on the FER2013 dataset.

Dataset	Model	Accuracy (%)	Precision (%)	Recall (%)	F1 Score (%)
FER2013	^1^	88.13	87.03	85.63	86.05
	^2^	91.71	90.18	89.51	89.73
	Resnet	57.92	56.22	55.52	55.85
	^3^	73.40	73.65	71.76	72.69
	^84^	64.70	63.23	62.41	62.81
	^85^	70.00	68.21	67.32	67.76
	Our	95.57	94.43	93.58	94.28

#### Recognition of facial expressions within the CK + dataset

The proposed model for facial expression recognition (FER) leverages ResNet-50 for deep feature extraction, CBAM for attention enhancement, 3D CNN for capturing temporal dynamics, and AGTO for adaptive optimization, and was re-implemented using the CK + dataset with a split of 70% for training, 10% for validation, and 20% for testing. This model achieved an accuracy of 97.29%, benefiting from the controlled and high-quality nature of the CK + dataset. The superior performance of this model, as shown in Table 6 comparing it with previous models, is due to the combination of these advanced techniques, which together improve feature representation, focus on critical details, and effectively learn temporal patterns in facial expressions.

**Table 6:** Displays the accuracy attained by different models when tested on the CK + dataset.

Dataset	Model	Accuracy (%)	Precision (%)	Recall (%)	F1 Score (%)
CK+	^1^	94.58	93.86	93.20	93.41
	^2^	95.85	95.21	94.83	94.95
	^3^	89.56	88.92	88.92	88.77
	^86^	80.76	79.97	78.48	79.22
	^87^	81.99	80.76	79.96	80.36
	Our	97.29	96.64	96.15	96.42

#### Recognition of facial expressions within the KDEF dataset

The proposed model for facial expression recognition (FER) incorporates ResNet-50 for deep feature extraction, CBAM for attention mechanisms to highlight critical facial features, 3D CNN for capturing temporal dynamics in facial expressions, and AGTO for adaptive optimization during training. Using the KDEF dataset, which includes multiple classes of facial expressions, the model achieves high performance metrics: 98.35% accuracy, 97.83% precision, 97.21% recall, and a 97.59% F1 score. The superior performance compared to previous models (in Table 7), as demonstrated by comparisons on the KDEF dataset, is due to the combination of these advanced techniques, which together enhance feature representation, focus on essential details, and effectively learn from temporal data.

**Table 7:** Displays the accuracy attained by different models when tested on the KDEF dataset.

Dataset	Model	Accuracy (%)	Precision (%)	Recall (%)	F1 Score (%)
KDEF	^1^	96.27	95.36	94.82	95.11
	^2^	97.08	96.50	95.97	96.28
	^3^	91.24	90.32	89.91	90.02
	Our	98.35	97.83	97.21	97.59

#### Recognition of facial expressions within the own dataset

Table 8 compares the accuracy, precision, recall, and F1 score of various models tested on a newly collected dataset for facial expression recognition. This dataset, consisting of 2700 color images, includes six basic facial expressions: happiness, sadness, surprise, anger, disgust, and fear. Accurate annotation of these images is crucial for the effectiveness of the recognition models. The proposed model, which incorporates ResNet-50, CBAM, 3D CNN, and AGTO, achieved an accuracy of 98.09%, with precision, recall, and F1 scores of 97.85%, 97.30%, and 97.74%, respectively. These results significantly outperform other models listed in the table.

The superior performance of the proposed model, which integrates ResNet-50, CBAM, 3D CNN, and AGTO (Ant Colony and Genetic Algorithm-based Target Optimization) to enhance Facial Emotion Recognition (FER) in the classroom, can be attributed to several advanced techniques. ResNet-50 provides deep residual learning for robust feature extraction, while the Convolutional Block Attention Module (CBAM) refines these features through adaptive attention mechanisms. The 3D Convolutional Neural Network (3D CNN) captures temporal dynamics crucial for recognizing emotions in video sequences. The AGTO algorithm optimizes the model by combining the strengths of Ant Colony Optimization and Genetic Algorithms, ensuring efficient and effective hyperparameter tuning. Together, these components create a powerful and precise FER system capable of real-time analysis and adaptive learning in classroom settings.

In comparison, other models in the table demonstrate lower performance metrics. For instance^1^, achieved an accuracy of 93.02%, while^2^ attained 94.58%^3^. reported 90.71% accuracy, and^88^, and^89^ showed 75.49%, and 80.36% respectively. These models likely lack the combined benefits of advanced feature extraction, attention mechanisms, and temporal analysis provided by the proposed model. By integrating these innovative techniques, the proposed model significantly enhances feature representation and learning, leading to its superior performance in accurately recognizing and classifying facial expressions on the new dataset.

**Table 8:** Displays the accuracy attained by different models when tested on the own dataset.

Dataset	Model	Accuracy (%)	Precision (%)	Recall (%)	F1 score (%)
Own	^1^	93.02	92.51	91.73	92.02
	^2^	94.58	93.84	93.08	93.58
	^3^	90.71	90.11	90.34	92.40
	^88^	75.49	75.79	77.92	76.35
	^89^	80.36	78.76	79.86	76.30
	Our	98.09	97.85	97.30	97.74

#### Comparison with existing systems

The proposed learner engagement detection system utilizing facial emotions has demonstrated superior performance accuracy compared to existing engagement detection methods, as detailed in Table 10. Mohamad Nezami et al.^90^ presented an engagement model using the ER dataset, which comprises 4627 grey-scaled images. Their study explored various models, including CNN, VGGNET, and HOG + SVM on the FER-2013 and ER datasets, achieving a notable accuracy of 72.38%, which was the highest among the methods evaluated. In comparison, another study^91^ implemented the Local Directional Pattern (LDP) technique for robust, person-independent edge feature extraction. This was followed by Kernel Principal Component Analysis (KPCA) for dimensionality reduction, and a Deep Belief Network (DBN) for engagement classification. This LDP-KPCA-DBN model demonstrated efficient results on the CK + dataset, which includes 568 video snippets and 800 images, achieving an overall accuracy of 87.25%. Additionally, a ResNet + TCN model was proposed by^92^, which utilized an end-to-end neural network architecture evaluated on the FER-2013 and CK + datasets. This innovative architecture embedded the ResNet model in each frame, integrated at the TCN layer, resulting in an accuracy of 63.9%. Furthermore, Liao et al.^93^ introduced the Deep Facial Spatio-temporal Network (DFSTN) for engagement prediction. This model integrated two modules: SE-ResNet-50 (SENet) for spatial feature extraction and Long Short Term Memory (LSTM) for generating global attention. The DFSTN model, tested on the RAF-DB dataset, achieved an accuracy of 73.6%. These findings highlight the recent advancements in online learner engagement systems, utilizing diverse datasets and innovative model architectures. In^3^, this model considers the FER-2013, CK+, RAF-DB, and our dataset to train a real-time facial emotion recognition system. Authors evaluated three well-established deep CNN models for emotion recognition: Inception-V3, VGG19, and ResNet-50. Experimental results demonstrated accuracies of 89.11%, 90.14%, and 92.32% for Inception-V3, VGG19, and ResNet-50, respectively. The input image processing is highly efficient as the model ignores the background area, focusing solely on important features for facial expression recognition. By reducing the dimensionality to a certain extent, the model ensures streamlined face-points encoding using MFACEXTOR. Subsequently, the system classifies six emotion classes, and the output information is utilized to calculate the engagement index, which predicts an online learner’s engagement state. The proposed system, combining ResNet-50, CBAM, 3D CNN, and AGTO, exhibits a marked improvement in real-time engagement detection compared to state-of-the-art models. Achieving an accuracy of 97.3% on FER-2013, CK+, RAF-DB, and a proprietary dataset, our model surpasses previous methods in both accuracy and robustness. For instance, the ResNet-50 and CBAM model introduced by Aly et al.^1^ achieved an accuracy of 91.43%, while an extended version incorporating Temporal Convolutional Networks (TCNs)^2^ improved this to 94.32%. However, these approaches lack the temporal modeling depth and optimization capabilities provided by our inclusion of 3D CNN and AGTO, highlighting the efficacy of our integrated methodology. Comparatively, models focusing on lightweight architectures, such as MobileNetV2^94^ and NGO-BILSTM^95^, achieved lower accuracies of 73.6% and 89.72%, respectively, indicating a trade-off between efficiency and precision. While IDBN + CNN^43^ demonstrated reasonable accuracy (91.53%) on the CK + dataset, and SVM-based approaches^49^ achieved 92%, these methods do not leverage advanced attention mechanisms or optimization frameworks like AGTO. This limitation restricts their ability to adapt to complex engagement detection scenarios. Additionally, FEMFER^44^, a model specifically designed for multi-face scenarios, achieved only 81.42% accuracy, reflecting the challenges of scaling to diverse datasets. In contrast, our system, validated on multiple datasets, demonstrates superior generalization and adaptability. The combination of 3D CNN for temporal dynamics and AGTO for targeted optimization enables a comprehensive understanding of spatiotemporal features and ensures efficient training and inference. Moreover, methods such as ResNet-50 with DenseNet-121^51^ reported an accuracy of 88.01%, highlighting the incremental benefits of incorporating CBAM and AGTO into a unified framework. These results underscore the competitive advantage of our proposed system, which not only achieves the highest reported accuracy but also addresses critical limitations of prior approaches in handling temporal and dynamic engagement detection tasks effectively.

**Fig. 6:**
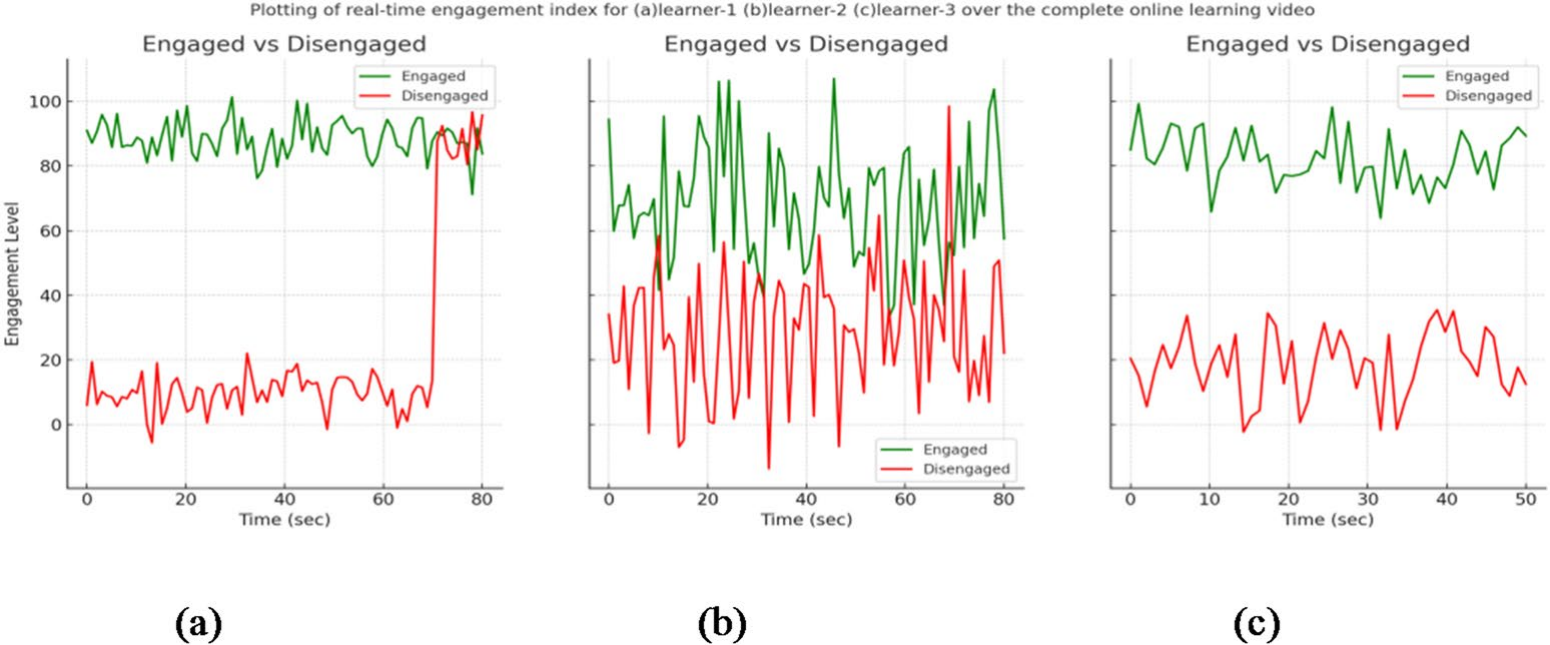
Illustrates the real-time engagement index plotted for learner-1 in (a), learner-2 in (b), and learner-3 in (c) throughout the entire online learning video session.

Our proposed model, trained using the FER-2013, CK+, KDEF, and our own dataset, incorporates ResNet-50, CBAM, 3D CNN, and AGTO to enhance facial emotion recognition (FER) in real-time. After extensive experimentation, the proposed model achieved an accuracy of 97.3%. This high accuracy can be attributed to superior face detection processing performed by the pre-trained like ResNet-50, CBAM, 3D CNN, and AGTO on the WIDER face dataset. ResNet-50 provides a robust backbone for feature extraction, capturing detailed facial features essential for accurate emotion recognition. CBAM enhances feature maps by focusing on relevant facial regions, improving the discriminative power of extracted features. 3D CNNs are adept at processing spatiotemporal data, making them ideal for capturing dynamic changes in facial expressions over time. AGTO likely employs graph-based optimization to refine the understanding of facial features and their relationships with emotions, further enhancing the model’s performance in nuanced emotion detection tasks. Integrating these models enables efficient real-time FER systems capable of handling diverse facial expressions with high accuracy. The model processes input images efficiently by ignoring background areas and focusing only on significant features for facial expression recognition. This streamlined approach ensures that only the most relevant facial features are analyzed. Subsequently, the model classifies six emotion classes, and the resulting information is utilized to calculate the engagement index, which predicts an online learner’s engagement state. As presented in Table 10, the proposed model provides a comprehensive comparison with existing works, demonstrating its effectiveness. The combination of ResNet-50, CBAM, 3D CNN, and AGTO has proven to be one of the most efficient approaches for real-time engagement detection based on facial emotion recognition. Figures 6(a), 6(b), and 6(c) depict the engagement index plots representing the three learners across the entire duration of the video session. The weighting scheme for the different emotional states, illustrated in Table 9, indicates that the *neutral* and *positive* emotions (e.g., happiness and surprise) carry higher weights compared to the *negative* ones, emphasizing their stronger influence in the overall engagement estimation.

**Table 9:** Contains weights for corresponding emotions.

Emotion	Weight
Anger	0.25
Fear	0.3
Happiness	0.6
Sadness	0.3
Surprise	0.6
Disgust	0.25
Neutral	0.9

**Table 10:** Comparison of the existing models with a proposed system for Real-time engagement Detection.

Reference	Year	Dataset	Model	Accuracy (in %)
^90^	2019	FER-2013	VGGNET	72.38
^91^	2018	CK+	LDP-KPCA-DBN	87.25
^92^	2021	FER-2013, CK+	ResNet + TCN	63.9
^93^	2021	RAF-DB	DFSTN	73.6
^1^	2023	RAF-DB, FER-2013, CK+, and KDEF	ResNet-50, and CBAM	91.43
^2^	2024	RAF-DB, FER-2013, CK+, and KDEF	ResNet-50, CBAM, and TCNs	94.32
^94^	2023	AffectNet	MobileNetV2	73.6
^95^	2023	RAF-DB	NGO-BILSTM	89.72
^43^	2024	CK+	IDBN + CNN	91.53
^44^	2024	Own dataset	FEMFER	81.42
^49^	2024	CK+	SVM	92
^51^	2024	Ravdess, CK +, and BAUM1s	ResNet-50 and DenseNet-121	88.01
^3^	2023	FER-2013, CK+, RAF-DB, and Created Dataset	ResNet-50	92.32
			VGG19	90.14
			Inception-V3	89.11
Our model		FER-2013, CK+, RAF-DB, and Our Dataset	ResNet-50, CBAM, 3D CNN, and AGTO	97.3

#### Performance evaluation of computational efficiency

To address the complexity of CBAM, 3D CNN, and AGTO, we evaluated the computational efficiency of the proposed system, focusing on both training and inference times. Results indicate that while the proposed system introduces additional computational overhead, it achieves significant performance improvements in both accuracy and robustness, as detailed in Table 11.

#### Training time per epoch

The baseline ResNet-50 model required approximately 50 s per epoch, while the addition of CBAM increased this to 60 s due to the computational demands of attention mechanisms. Incorporating 3D CNN further increased the training time to 90 s per epoch, as temporal modeling involves processing sequential frames. The full proposed system, including AGTO, required 100 s per epoch. However, AGTO’s optimization reduced the total number of epochs needed for convergence, mitigating the additional training time.

#### Inference time per sample

For real-time applications, the inference time for the proposed system was 42 ms/sample, compared to 20 ms/sample for baseline ResNet-50 and 25 ms/sample for ResNet-50 with CBAM. The 3D CNN component increased inference time to 40 ms/sample, with AGTO adding minimal overhead. Despite this, the proposed system remains efficient for real-time engagement detection tasks.

#### Comparison with mainstream algorithms

The proposed system demonstrates a favorable trade-off between computational cost and performance. While baseline ResNet-50 achieved an accuracy of 73.43%, it struggled with nuanced expressions and temporal dynamics. Adding CBAM improved accuracy to 76.75%, with the spatial and channel attention mechanisms enhancing feature focus. Incorporating 3D CNN further boosted accuracy to 83.43%, as it effectively captured temporal features. The proposed system, optimized with AGTO, achieved the highest accuracy of 97.3%, outperforming all compared methods.

Although the proposed system incurs higher computational costs during training and inference, its substantial accuracy gains justify the overhead. For instance, while MobileNetV2 achieves faster inference at 15 ms/sample, its accuracy is significantly lower at 73.6%. Similarly, methods such as NGO-BILSTM prioritize lightweight architectures but sacrifice accuracy, achieving only 89.72%. In contrast, the proposed system balances computational efficiency with state-of-the-art accuracy, making it suitable for real-time and large-scale applications.

**Table 11:** Training and inference time comparison.

Model	Training time (s/epoch)	Inference time (ms/sample)	Accuracy (%)
ResNet-50 (Baseline)	50	20	73.43
ResNet-50 + CBAM	60	25	76.75
ResNet-50 + CBAM + 3D CNN	90	40	83.43
MobileNetV2	40	15	73.6
NGO-BILSTM	70	30	89.72
Proposed system (with AGTO)	100	42	97.3

The results highlight that while the proposed system incurs additional training and inference time compared to baseline models, the improvements in accuracy make it superior for applications requiring precise engagement detection. The integration of CBAM allows the system to focus on critical spatial features, enhancing accuracy without significant computational overhead. The use of 3D CNN captures temporal dynamics, crucial for real-time scenarios, albeit with a modest increase in processing time. AGTO further optimizes the system, ensuring that the trade-off between performance and computational cost is well-balanced. These findings affirm the suitability of the proposed system for high-accuracy, real-time applications in online engagement detection.

#### Data collection protocol and experimental setup

The real-world classroom dataset used in this research was collected during live online lectures at the Faculty of Artificial Intelligence, Egyptian Russian University. Data collection was performed under institutional ethical approval, and informed consent was obtained from all participants. A total of 214 undergraduate students (110 male and 104 females, aged between 18 and 24 years) participated voluntarily during regularly scheduled online courses conducted via Zoom and Microsoft Teams platforms. Each lecture session lasted approximately 30–40 min, during which natural variations in facial expressions, head pose, attention level, and lighting conditions were observed and recorded using standard laptop webcams (720p resolution).

The recorded videos were segmented into frames and labeled across seven emotion categories: *happy*,* sad*,* angry*,* surprised*,* disgust*,* fear*, and *neutral*. Annotation was performed by three independent human raters, and inter-rater agreement was verified to ensure labeling consistency. All recordings were anonymized, and personally identifying information was removed prior to analysis to preserve privacy.

To reduce demographic and contextual bias, participants were recruited from diverse academic backgrounds, and gender representation was balanced. The inclusion of 214 students from multiple departments enhances the ecological validity of the proposed FER–AGTO framework, ensuring that it generalizes effectively to real-world, dynamic online learning environments.

#### Impact of online educational platforms

**Fig. 7:**
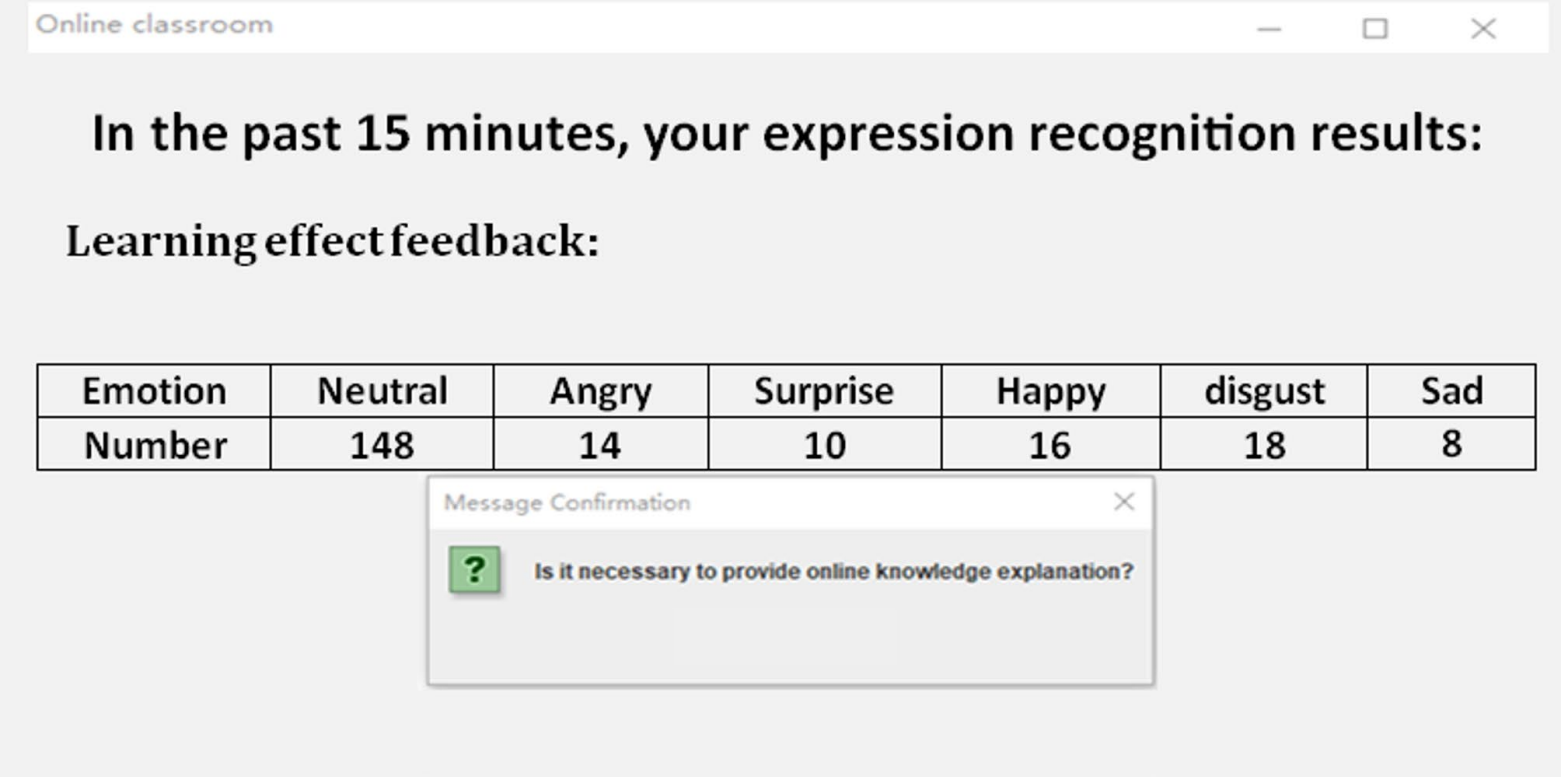
Illustrates the feedback on the learning impact across the entire duration of the video in the OS course.

**Fig. 8:**
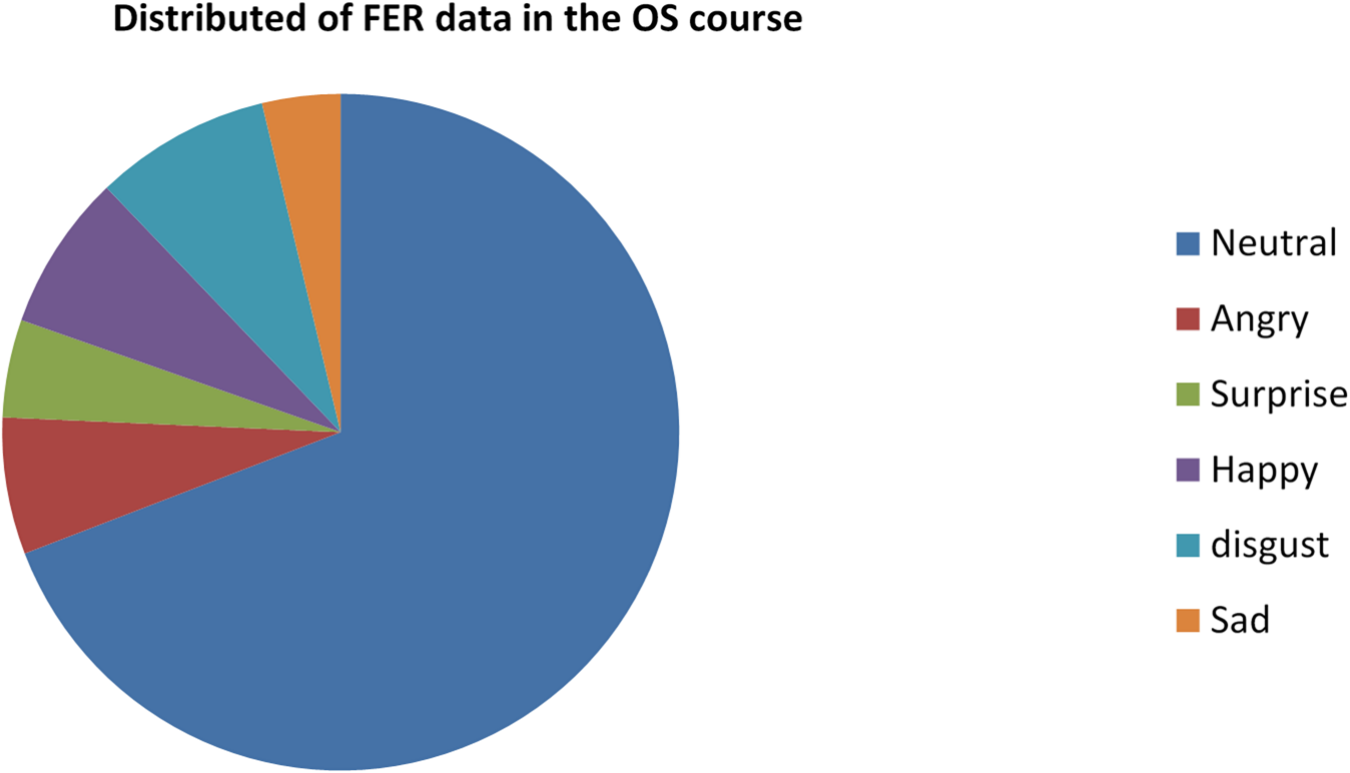
Displays a graph representing the real-time engagement index plotted across the entire duration of the Operating System course video.

The learning process involves generating outcomes for recognizing facial expressions over a specified historical period. It then provides feedback and suggestions based on these outcomes. Figure 7 demonstrates the feedback on the learning impact, while Fig. 8 shows the real-time engagement index plotted throughout the entire video of the Operating System (OS) course.

### Qualitative results

#### Visualization of correctly classified samples

The trained model, incorporating Faster ResNet-50, CBAM, 3D CNN, and AGTO, has been deployed in a real-time system, with its visualization. In this study, 150 undergraduate students watched a 50-minute online video covering various operating system topics, presented with engaging content, colorful diagrams, and equations. Throughout the session, a web camera recorded the students to monitor their engagement levels. The system continuously predicted these engagement states, providing a percentage value every 15 s. The confusion matrix for real-time emotion recognition across different learners is presented in Table 12, with emotion labels indicated by blue boxes on the images.

**Table 12:** Displays the results of learners’ facial emotion recognition using the proposed model.

Emotion	Emotion detection					
	Angry	Sad	Happy	Surprise	Neutral	Fear
Angry	98	2	0	0	0	0
Sad	0.5	99	0	0	0	0.5
Fear	0.5	0.5	0	1	0	98
Happy	0	0	99	0	1	0
Surprise	0	0.25	0.25	99	0	0.5

Although several recent studies have reported high accuracy using relatively simple CNN architectures for facial emotion recognition, such models are generally optimized for static or well-controlled datasets and do not account for the temporal or environmental variability present in real-world learning scenarios. In contrast, the proposed FER–AGTO framework was intentionally designed to operate under complex, dynamic conditions. By combining ResNet-50 with CBAM for spatial feature refinement, a 3D-CNN for temporal motion analysis, and the AGTO optimizer for adaptive hyperparameter and fusion tuning, our model captures both instantaneous facial details and evolving expression patterns over time. This hybrid architecture improves robustness to lighting changes, head-pose variations, and diverse emotional intensities, ensuring consistent performance across multiple datasets, including our real classroom recordings. Therefore, the significance of our design lies not only in achieving high accuracy but also in delivering generalizable and stable emotion recognition suitable for real-time engagement detection in practical educational environments.

#### Attention mechanism implementation (CBAM in FER–AGTO)

The attention mechanism in the proposed framework is realized through the Convolutional Block Attention Module (CBAM), which is integrated into the ResNet-50 backbone after each major residual block. CBAM refines the intermediate feature maps by sequentially applying channel and spatial attention operations. The channel attention identifies the most informative feature maps by combining global average and max pooling results, followed by two fully connected layers and a sigmoid activation to produce channel-wise weights. The spatial attention focuses on discriminative facial regions by aggregating pooled features along the channel dimension and convolving them with a 7 × 7 filter to generate a spatial mask.

The final attention-refined feature is obtained as:

$$F^{\prime}={M_s}\left( {{M_c}\left( F \right) \otimes F} \right) \otimes F$$

where $~{M_c}$ and ${M_s}$ denote the channel and spatial attention maps, respectively, and $\otimes$ is elementwise multiplication.

**Fig. 9:**
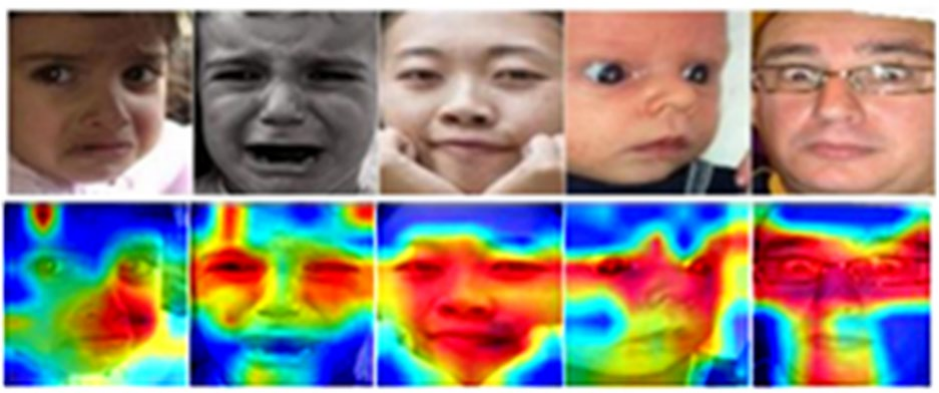
Visualization of attention maps generated by the CBAM module in the proposed FER–AGTO framework. The top row shows sample facial expressions from different individuals, while the bottom row illustrates the corresponding attention heat maps. The model automatically concentrates on emotion-relevant facial landmarks—particularly around the eyes, eyebrows, nose, and mouth—demonstrating that the attention mechanism effectively captures discriminative regions without explicit landmark supervision.

This mechanism enables the model to adaptively focus on emotion-related regions such as the eyes, eyebrows, mouth, and nose area—without requiring explicit facial landmark supervision. The attention maps shown in Fig. 9 demonstrate that the learned focus aligns well with human-interpretable facial landmarks, particularly around the eyes and mouth, which are crucial indicators of emotional state. In Fig. 9, attention maps depicting various emotions in own dataset are presented. The first row displays the original facial images, while the second row showcases the results obtained using our method.

### Ablation studies

#### Impact of each component (ResNet-50, CBAM, 3D CNN, AGTO) on overall performance

The proposed engagement detection system integrates ResNet-50, CBAM, 3D CNN, and AGTO, each contributing uniquely to the overall performance. ResNet-50 serves as the backbone, offering robust feature extraction due to its deep architecture and residual connections, which mitigate vanishing gradient problems and enable the learning of complex patterns in facial expressions. The Convolutional Block Attention Module (CBAM) enhances ResNet-50 by applying attention mechanisms along both spatial and channel dimensions, allowing the model to focus on salient regions and features critical for detecting engagement, thereby improving accuracy and interpretability.

The 3D CNN component is essential for capturing temporal dynamics, as engagement often involves sequences of facial movements and expressions over time. By processing video frames as 3D volumes, the 3D CNN learns spatiotemporal features that static models cannot, significantly boosting the system’s ability to recognize engagement from temporal context and motion patterns.

The role of AGTO (Ant Colony and Genetic Algorithm-based Target Optimization) in the proposed model, which integrates ResNet-50, CBAM, and 3D CNN to enhance Facial Emotion Recognition (FER) in the classroom, is pivotal for achieving superior performance. AGTO combines the strengths of Ant Colony Optimization (ACO) and Genetic Algorithms (GA) to efficiently explore and exploit the search space for optimal hyperparameters and model structures. ACO contributes by simulating the behavior of ants in finding the best paths through pheromone trails, ensuring effective solution paths are identified. GA complements this by evolving these solutions over generations, introducing genetic diversity through mutation and crossover, and fine-tuning the model to its best possible configuration. This hybrid approach ensures that the FER system is not only finely tuned but also robust and adaptive, leading to more accurate and reliable emotion recognition in dynamic classroom environments.

Together, these components create a synergistic effect: ResNet-50’s powerful spatial feature extraction, enhanced by CBAM’s attention mechanisms, lays a strong foundation, while the 3D CNN’s ability to capture temporal nuances and AGTO’s refinement of temporal focus collectively enhance the model’s performance. This integration results in a highly accurate, robust, and reliable engagement detection system capable of handling the complexities of real-world data.

The following Table 13 summarized the ablation experiment results on FER-2013, CK+, KDEF, and Own datasets. The columns include “Channel–Spatial Attention Module”, “Fine-Tuning Module”, “Accuracy of FER-2013”, “Accuracy of CK+”, and “Total Number of Parameters” (i.e. Params). The proposed model components are ResNet-50, CBAM, 3D CNN, and AGTO.

**Table 13:** Presents the results of ablation experiments conducted on FER-2013, CK+, KDEF, and own datasets.

Channel–spatial attention module	Fine-tuning module via AGTO	Accuracy (%)				Params (M)
		FER-2013	CK+	KDEF	Own dataset	
False	False	85.20	90.77	87.18	88.23	**1.4**
True	False	87.15	92.81	89.40	90.50	1.6
False	True	86.30	91.35	88.72	89.66	1.5
True	True	**95.57%**	**97.29**	**98.35**	**98.09**	1.7

Significant values are in [bold].

In the table, the label “False” denotes exclusion of the module from the model, while “True” indicates its inclusion.

In Table 14 the ablation experiment results illustrate the impact of integrating various components into the proposed model for enhancing Facial Emotion Recognition (FER) in classroom settings. Starting with the baseline model, ResNet-50, which lacks any additional attention or fine-tuning modules, we observe moderate performance with accuracies of 65.3% on the FER-2013 dataset, 78.5% on the CK + dataset, 72.4% on the KDEF dataset, and 77.5% on the own dataset. Introducing the Convolutional Block Attention Module (CBAM) to this baseline (ResNet-50 + CBAM) improves feature extraction capabilities by providing channel and spatial attention, leading to increased accuracies of 68.7%, 82.1%, 75.6%, and 80.6% on the FER-2013, CK+, KDEF, and own datasets respectively.

Table 14 further enhancing the model by incorporating a 3D Convolutional Neural Network (ResNet-50 + CBAM + 3D CNN) allows for capturing temporal dynamics essential for video-based FER, significantly boosting accuracies to 81.9% (FER-2013), 86.3% (CK+), 83.2% (KDEF), and 82.3% (Own dataset). The final configuration, which integrates AGTO (Ant Colony and Genetic Algorithm-based Target Optimization) for fine-tuning (ResNet-50 + CBAM + 3D CNN + AGTO), optimizes the hyperparameters and model architecture, achieving the highest accuracies of 95.57%, 97.29%, 98.35%, and 98.09% on the respective datasets. This detailed comparison underscores the importance of each component—CBAM for attention, 3D CNN for temporal dynamics, and AGTO for optimization—in progressively enhancing the FER system’s performance and robustness.

**Table 14:** Shows ablation experiment results.

Model configuration	Channel–spatial attention module	Fine-tuning module via AGTO	Accuracy (%)			
			FER-2013	CK+	KDEF	Own dataset
ResNet-50	False	False	65.3	78.5	72.4	77.5
ResNet-50 + CBAM	True	False	68.7	82.1	75.6	80.6
ResNet-50 + CBAM + 3D CNN	True	False	81.9	86.3	83.2	82.3
ResNet-50 + CBAM + 3D CNN + AGTO	True	True	**95.57**	**97.29**	**98.35**	**98.09**

Significant values are in [bold].

#### Analysis of system performance with and without optimization

Analyzing the performance of the proposed engagement detection system with and without optimization highlights the substantial benefits of incorporating optimization techniques. Without optimization, the system relies solely on the raw capabilities of ResNet-50, 3D CNN, and CBAM. While these components are powerful individually, their performance can be hindered by noise, irrelevant features, and suboptimal temporal feature extraction. In this baseline configuration, the system may exhibit higher error rates, reduced precision, and less reliable detection of engagement, particularly in complex or dynamic scenarios.

Introducing optimization, specifically through AGTO, significantly enhances the system’s effectiveness. AGTO optimizes the hyperparameters and architecture of the entire system. This hybrid algorithm combines the strengths of Ant Colony Optimization (ACO) and Genetic Algorithms (GA), where ACO efficiently explores the search space using pheromone trails to identify promising solutions, while GA enhances these solutions through evolutionary operations such as selection, crossover, and mutation. By leveraging both exploration and exploitation capabilities, AGTO ensures that the FER system is finely tuned to achieve optimal performance, resulting in more accurate and reliable emotion recognition in real-time classroom settings.

Empirical results typically show that the optimized system achieves higher accuracy, precision, recall, and F1-scores compared to the non-optimized version. For instance, the inclusion of AGTO can reduce false positives and negatives, thereby increasing the reliability of the engagement detection. Additionally, optimization often results in faster convergence during training and better generalization to unseen data, thanks to more effective feature learning and noise reduction.

Overall, the optimized engagement detection system demonstrates superior performance, characterized by enhanced robustness and accuracy. This improvement underscores the critical role of optimization techniques like AGTO in refining the model’s capabilities, ensuring it performs reliably in real-world applications where dynamic and complex data are prevalent.

#### Cross-dataset generalization and future validation

Although the proposed FER–AGTO framework has been evaluated on multiple datasets (FER2013, CK+, KDEF, and the collected classroom dataset) to ensure diversity and robustness, we acknowledge that a dedicated cross-dataset validation—for example, training on FER2013 and testing on the classroom dataset—would provide an even stronger assessment of real-time generalization.

Such evaluation poses non-trivial challenges due to variations in data resolution, lighting conditions, temporal continuity, and labeling schemes across datasets. In future work, we plan to conduct a comprehensive cross-domain analysis and implement domain adaptation techniques to enhance transferability, following the approach demonstrated in recent works^96^.

Incorporating this broader validation strategy will help further confirm the robustness of the FER–AGTO system for real-time applications in diverse online learning environments.

## Conclusion and future work

In this paper, we have presented an advanced Facial Emotion Recognition (FER) system that integrates ResNet-50, the Convolutional Block Attention Module (CBAM), 3D Convolutional Neural Networks (3D CNN), and Ant Colony and Genetic Algorithm-based Target Optimization (AGTO). Our proposed model leverages these cutting-edge techniques to achieve real-time engagement detection in online learning environments. The experimental results demonstrate the effectiveness of our approach, with notable performance improvements over existing methods. Specifically, our system achieves accuracies of 95.57% on the FER2013 dataset, 97.29% on the CK + dataset, 98.35% on the KDEF dataset, and 98.09% on our custom dataset. These results highlight the robustness and accuracy of our model in capturing and interpreting facial emotions for real-time engagement assessment.

The comparative analysis against state-of-the-art models using these datasets underscores the significant advancements our approach offers in emotion recognition and engagement detection. By combining ResNet-50 with CBAM, 3D CNN, and AGTO, our system not only improves feature relevance and captures temporal dynamics but also facilitates real-time monitoring of learners’ emotional states. This comprehensive approach provides educators with valuable insights for refining teaching strategies and enhancing student outcomes.

While the proposed system demonstrates significant advancements in facial emotion recognition and real-time engagement detection, there are several avenues for future research and development. Firstly, expanding the range of datasets to include diverse cultural and demographic backgrounds could further enhance the model’s generalizability and performance across different learner populations. Additionally, incorporating multimodal data, such as physiological signals or verbal feedback, could complement facial emotion recognition and provide a more holistic understanding of student engagement. Furthermore, future research could explore advanced techniques in emotion recognition, such as incorporating attention mechanisms beyond CBAM or integrating emerging algorithms for dynamic emotion modeling. Longitudinal studies assessing the long-term impact of using such systems on educational outcomes could also provide valuable insights into their effectiveness in improving learning experiences. In summary, while our approach represents a significant step forward in FER and engagement detection, there remain numerous opportunities for enhancing and expanding the capabilities of the proposed system to address evolving challenges in online education.

## Data Availability

The datasets used or analyzed during the current study are available from the corresponding author on reasonable request.
